# Effects of Exercise Training on the Autonomic Nervous System with a Focus on Anti-Inflammatory and Antioxidants Effects

**DOI:** 10.3390/antiox11020350

**Published:** 2022-02-10

**Authors:** Matei Daniela, Luca Catalina, Onu Ilie, Matei Paula, Iordan Daniel-Andrei, Buculei Ioana

**Affiliations:** 1 Department of Biomedical Sciences, Faculty of Medical Bioengineering, University of Medicine and Pharmacy “Grigore T. Popa” Iasi, 700454 Iasi, Romania; daniela.matei@umfiasi.ro (M.D.); buculei.ioana@umfiasi.ro (B.I.); 2 Doctoral School of Faculty of Chemical Engineering and Environmental Protection “Cristofor Simionescu”, Technical University “Gheorghe Asachi” Iasi, 700050 Iasi, Romania; 3 Faculty of General Medicine, University of Medicine and Pharmacy “Grigore T. Popa” Iasi, 700115 Iasi, Romania; mg-rom-30171@students.umfiasi.ro; 4 Department of Individual Sports and Kinetotherapy, Faculty of Physical Education and Sport, “Dunărea de Jos” University of Galati, 800008 Galati, Romania; daniel.iordan@ugal.ro

**Keywords:** autonomic nervous system, sympathetic, parasympathetic, immunity, oxidative stress, physical exercise

## Abstract

Studies show that the autonomic nervous system (ANS) has an important impact on health in general. In response to environmental demands, homeostatic processes are often compromised, therefore determining an increase in the sympathetic nervous system (SNS)’s functions and a decrease in the parasympathetic nervous system (PNS)’s functions. In modern societies, chronic stress associated with an unhealthy lifestyle contributes to ANS dysfunction. In this review, we provide a brief introduction to the ANS network, its connections to the HPA axis and its stress responses and give an overview of the critical implications of ANS in health and disease—focused specifically on the immune system, cardiovascular, oxidative stress and metabolic dysregulation. The hypothalamic–pituitary–adrenal axis (HPA), the SNS and more recently the PNS have been identified as regulating the immune system. The HPA axis and PNS have anti-inflammatory effects and the SNS has been shown to have both pro- and anti-inflammatory effects. The positive impact of physical exercise (PE) is well known and has been studied by many researchers, but its negative impact has been less studied. Depending on the type, duration and individual characteristics of the person doing the exercise (age, gender, disease status, etc.), PE can be considered a physiological stressor. The negative impact of PE seems to be connected with the oxidative stress induced by effort.

## 1. Introduction

The autonomic nervous system (ANS) has been identified as playing an important role in affecting general health. In modern societies, chronic stress associated with an unhealthy lifestyle contributes to ANS dysfunction, which is involved in the pathogenesis of neuroendocrine, cardiovascular, respiratory, digestive and psychiatric disorders.

Physical exercise has been shown to have a positive impact on health in general, but the negative impact of high intensity exercise is less studied. The oxidative stress caused by these types of physical training has an important influence on the ANS.

In this review, we first provide a brief introduction to the ANS network and its connections to the hypothalamic–pituitary–adrenal axis. Then, we give an overview of the critical implications of ANS in health and disease, focused specifically on inflammation and oxidative stress.

The methodological approach used for the present review is a systematic meta-analysis. The aim of this article is to try to demonstrate the benefits of physical exercise on oxidative stress and inflammation and thus to ensure the maintenance of a harmonious balance between the sympathetic and parasympathetic nervous systems.

## 2. State of the Art

### 2.1. Autonomic Nervous System Network

The ANS acts through two main systems: the sympathetic nervous system (SNS) and the parasympathetic nervous system (PNS).

Stimulation of the SNS brings the body to a state of increased activity called the “fight or flight” response (heart rate and blood pressure increase, temporary inhibition of the gastrointestinal tract peristalsis, etc.) [1]. Preganglionic and postganglionic sympathetic neurons facilitate communication between the SNS and the peripheral organs. The point at which the axon of the preganglionic neuron connects with the postganglionic neuron is also the place where the cell body of the preganglionic neuron can be found, between the segments of the first thoracic and third lumbar spinal cord. Subsequently, the postganglionic neuron axon reaches the target organ.

Norepinephrine (NE), epinephrine (E) and dopamine (DA) are neurotransmitters. In response to a stimulus, most sympathetic postganglionic neurons release NE, which activates the adrenergic receptors (ARs) located on the target organ. The ARs family includes three β (β1, β2, β3), three α1 (α1A, α1B, α1D) and three α2 (α2A, α2B, α2C) receptor subtypes [1]. The sympathetic endings of sweat glands and the arterioles of somatic muscles are cholinergic. Matsukawa et al. published the results of a study conducted on animals showing that hypothalamic stimulation produces the vasodilatation of small arteries (with an internal diameter of 50–500 µm) in skeletal muscle [2]. This vasodilation is thought to be the result of a neural mechanism through sympathetic cholinergic nerves [2].

NE adapts physiological processes like mood, arousal, learning and memory, blood flow and metabolism. DA plays a variety of roles in humans and is involved in movement, memory, behavior, cognition, pleasure, sleep and personality. Monoamine deficiency influences anxiety disorders, depression, drug addictions, bipolar disorders and Parkinson’s disease, while excess monoamine is noticed in schizophrenia.

The PNS regulates the body’s unconscious actions, which can be summarized as the “rest and digest” response, as these return the body functions back to normal: blood pressure lowers, heart rate slows down, intestinal and gland activity increases, sphincter muscles in the gastrointestinal tract relax, etc. [1].

The structures in which the parasympathetic preganglionic neurons can be found are the sacral spinal cord and the brainstem. The parasympathetic nuclei of the glossopharyngeal, oculomotor, vagus and facial nerves and the cardiac preganglionic neurons are located in the nucleus ambiguus.

The primary parasympathetic neurotransmitter is acetylcholine (Ach), which has been shown to regulate various processes such as arousal, learning and memory, cognition and modulation of sensory information [3]. The cholinergic receptors can be divided into two types, nicotinic (nAChRs), which are ion channels, or G protein-coupled muscarinic receptors (mAChRs) [3]. Neuronal nicotinic receptors are located at pre- and/ or post-synaptic sites in many cortical areas. The α7 nAChR subtype is highly expressed in regions of the brain involved in learning and memory, such as the hippocampus and the neocortex [4].

If the peripheral pathways of the ANS are relatively distinct and very well documented, the central control of the ANS involving several areas throughout the spine, bulbopontine, pontomesencephalic and forebrain is still discussed.

The lower brainstem level includes the nucleus of the solitary tract (NTS), the reticular formation of the ventrolateral and ventromedial medulla (VLM, VMM) and the parabrachial nucleus (PBN) and is involved in the reflex control of circulation, respiration, gastrointestinal function and micturition [5]. The upper brainstem level includes the periaqueductal gray matter (PAG), which integrates autonomic control with pain modulation and behavioral responses to stress [5]. The forebrain level includes the paraventricular and related nuclei of the hypothalamus, thalamus, amygdala, anterior cingulate, the insular and the medial prefrontal cortex, which are involved in the integration of autonomic and endocrine responses [1]. The anterior limbic circuit (insula, the anterior cingulate cortex, and amygdala) realize the integration of specific sensations with emotional and goal-related autonomic responses [1].

It is important to mention that all ANS afferent inputs overlap with the afferent pathways of nociceptors, thermo and muscle receptors; their complex integration ensures the integral functioning of the physical and mental functions as a whole [6].

The nucleus of the solitary tract (NST) is the relay station of taste, visceral afferent information and all afferent pathways that trigger medullary reflexes. These include the baroreflex, heart rate, arterial pressure reflex [7], carotid chemoreflex, pulmonary mechanoreflexes [8] and reflexes controlling the motility of the esophagus and stomach [9].

Noradrenergic neurons that innervate the hypothalamic paraventricular nucleus have their origin in the caudal NTS (A2 cell group). Projections from the cortex, limbic system and hypothalamus to the A2 cell group region provide a route through which emotional and cognitive events can modulate visceral responses to diverse threats [10].

The rostral ventrolateral reticular medulla (RVLM) is the principal area of the brainstem regulating respiratory and cardiovascular reflexes, which controls blood pressure by regulating vasoconstriction and cardiac output [11]. The caudal ventrolateral medulla (CVLM) is critical for baroreceptor reflex bradycardia [12]. CVLM neurons exert a sympathoinhibitory effect by inhibiting the RVLM barosensitive neurons [12]. The ventromedial medulla plays a key role in thermoregulation, vasoconstriction [13], pain modulation [14] and ventilation [15].

The raphe nuclei are connected to the suprachiasmatic nuclei, thus contributing to the circadian rhythm, and these neurons contain serotonin (5-hydroxytryptamine; 5-HT) [1]. This 5-HT contains 14 different receptor subtypes, which explains the variety of the actions of serotonin. For example, 5-HT transporter channel blockers are used in major depression, anxiety and panic disorders; 5-HT1A receptor agonists are used to treat anxiety; and 5-HT2A receptor antagonists are an important class of drugs for the treatment of schizophrenia.

The area postrema (AP) is a key player in the autonomic control of the cardiovascular system and the systems controlling appetite and metabolism [16]. The AP is a circumventricular organ because its endothelial cells do not contain tight junctions, which allows for the free exchange of molecules between blood and brain tissue [16]. The AP has chemoreceptors known to detect emetic agents in blood and acts as a vomit-inducing center [1].

Periaqueductal gray matter (PAG), also called the “central gray”, is the grey matter surrounding the mesencephalic aqueduct, which plays a major role in integrated autonomic and somatic responses to stress, pain modulation, and other adaptive functions [17]. The ventrolateral PAG plays an important role in nociceptive and autonomous afferent integration; the dorsolateral PAG contains the micturition center; the dorsolateral and lateral PAG are related to autonomic concomitants (tachycardia, hypertension) [17].

The parabrachial nuclei (PBN) are a relay for visceral, nociceptive and thermo receptive inputs [18]. Vestibular input into the PBN is related to motion sickness. Medial PBN mediate the baroreflex constriction of coronary vessels and PBN, with the Kolliker-Fuse nucleus being involved in respiratory rhythm generation [1].

The Barrington nucleus, also known as the pontine micturition center or pelvic organ stimulating center, is important for the coordination of the micturition reflex and the control of the lower gastrointestinal tract and sexual organ functions [19].

The locus coeruleus (LC) is the main source of norepinephrine in the brain [20] and plays an important role in behavioral arousal and hypothalamus pituitary axis stress-associated hyperactivity [21]. LC is connected to the paragigantocellularis nucleus, a major sympathoexcitatory brain region [22]. An increase in LC activity may lead to sleeplessness and impulsivity [23]. A chronic decrease in LC activity may be associated with limited emotionality and flat affect that are observed in depressed patients and in patients suffering from dementia [24].

The anterolateral group of the bed nucleus of the stria terminalis (alBST) is connected to autonomic-related portions of the hypothalamus and caudal medulla and receives an extremely dense NE innervation that arises from A2 and A1 neurons but not from the LC [25]. NE acts within the alBST to modulate behavioral, hormonal and conditioned emotional responses to stress [26].

The hypothalamus is the major homeostatic center of the brain. It regulates endocrine activity and controls glucose, lipid metabolism, food and water intake, body temperature, blood flow and composition; it also drives behaviors related to feeding, emotional responses, autonomic function control in relation to sleep, arousal and motivated behavior [18]. The rostral and caudal hypothalamus is responsible for the control of the ANS. The rostral controls parasympathetic activity while the caudal controls sympathetic activity. A connection is also established between the hypothalamic paraventricular nucleus and the parasympathetic preganglionic neurons, also between the sympathetic preganglionic neurons and the brainstem autonomic centers.

The amygdala provides affective or emotional value to incoming sensory information [27] and generates responses that include autonomic function modulation. The basolateral complex of the amygdala has reciprocal connections with cortical association areas and the hippocampus, thus participating in learning and conditioned responses to aversive stimuli (fear) [27]. The central nucleus of amygdala is connected with the hypothalamus, PAG and medullary autonomic nuclei and initiates autonomic, neuroendocrine and motor responses to emotions and stress [28]. Exaggerated fear responses and the persistence of traumatic memories is a result of amygdala hyperactivation [29].

The insular cortex, anterior cingulate cortex and amygdala are connected with the prefrontal cortex, which regulates decision-making and emotional behavior [30]. The insular cortex (IC) is the primary interoceptive cortex and integrates visceral, pain and temperature sensations [6]. The insular cortex is subdivided into the granular insular cortex (GI), the dysgranular insular cortex (DI) and the agranular insular cortex (AI) [31]. The AI is believed to participate in nociceptive and autonomic processing [6], while DI is involved in gustatory processing and GI plays an important role in visceral function modulation [6]. IC is believed to be the brain site that mediates feelings [32], empathy [33] and positive and negative emotional states [34]. The left anterior insula is activated predominantly by parasympathetic functions and the right anterior insula is activated by pathways associated with sympathetic functions [35]. The activation of the right insula correlates with mean arterial blood pressure and heart rate during a task or exercise [30]. In a recent study the dorsal anterior insula were found to be bilaterally associated with parasympathetic functions while the right ventral anterior insula showed sympathetic predominance extending to the frontal operculum [36].

The anterior cingulate cortex (ACC) integrates autonomic responses with behavioral arousal. The rostral ACC is involved in emotional processing and the caudal ACC is involved in cognitive processing [37]. Functional MRI studies show that the caudal ACC is activated during tasks that involve awareness and attention and is associated with an increase in sympathetic drive [30]. The rostral ACC becomes inactivated during such tasks [38] and is involved in the parasympathetic control of the heart [30].

The prefrontal cortex (PFC) coordinates autonomic and neuroendocrine functions with cognitive and affective processes. The dorsomedial PFC suppresses the HPA axis response to acute psychological stress, while the ventromedial PFC serves to activate the HPA axis [39]. The dorsomedial PFC shares connections with the primary motor and somatosensory cortices, premotor area and somatosensory association areas and coordinates goal-directed actions [40]. The ventromedial PFC (vmPFC) has reciprocal projections with subcortical limbic structures and contribute to the regulation of stress and emotionality [41]. Additionally, vmPFC has projections with subcortical cell groups that release hormones to the hypothalamic–pituitary–adrenal axis and neurotransmitters from the autonomic nervous system. The orbital region of the PFC has been demonstrated to be involved in social learning and affective stimuli. The dorsolateral PFC is important for working memory, decision-making, and behavior [1]. Researchers have found right lateralized activity in the PFC during sadness and left lateralized activity in the PFC during relaxation [42].

Dysfunction within the PFC can produce disturbances in cognitive performance, emotional responses, autonomic regulation, neurotransmission and neuroendocrine responses that are associated with stress disorders [41].

A recent meta-analysis on the central processing of autonomic functions found that sympathetic regulation involved the prefrontal cortex, the anterior and mid cingulate cortex and the right ventral anterior insular and left posterior insular cortices, while parasympathetic regulation involved the posterior cingulate cortex, the lateral temporal cortices, the bilateral dorsal anterior insula and hippocampal formation [36]. Regions that showed both sympathetic and parasympathetic functions included the left amygdala, the right inferior parietal lobule and a small area in the right anterior insula [36].

### 2.2. The Autonomic Nervous System and Hypothalamic–Pituitary–Adrenal Axis

Mental processes influence autonomic responses in order to alter the physical state of the body. Additionally, the internal physiological state of the body can influence mental processes.

Stress refers to a condition that can have an impact on a person’s physical or psychological wellbeing. Stress systems include the sympathetic–adrenal–medullary axis and the hypothalamic–pituitary–adrenal axis (HPA).

In response to biological stress, the first neurons activated are the NE neurons of the LC. Subsequently, the paraventricular nucleus (PVN) of the hypothalamus initiates corticotropin releasing hormone (CRH) secretion, thus inducing adrenocorticotropin hormone (ACTH) release, decreased secretion of gonadotropins, increased secretion of prolactin and growth hormone from the anterior pituitary glands, increased secretion of renin from the kidneys and pancreatic glucagon secretion [43]. ACTH stimulates the release of glucocorticoids (cortisol in humans) from the adrenal cortex into general circulation. The adrenal secretion of cortisol is modulated by a negative feedback mechanism involving the central nervous system, hypothalamus and pituitary and adrenal glands [44].

The amygdala activates the HPA axis, whereas the hippocampus and prefrontal cortex inhibit the HPA axis [28]. HPA hyperactivity is frequent in major depressive disorder, whereas reduced activity of the HPA axis is found in atypical depression.

Psychological stress (“anticipatory” stress) can be defined as a discrepancy between personal capacities and environmental requirements. In this situation, the organism only anticipates a threat to homeostasis based on prior experience [45]. Psychological stressors depend on corticolimbic structures such as the amygdala, hippocampus and PFC, which are responsible for discerning between threatening and non-threatening stimuli [28]. An emotional or anticipated stress response can happen without a primary sensory stimulus because limbic brain regions will stimulate the PVN when activated.

Both acute and chronic stress may be detrimental to health. Acute stress is a normal adaptive reaction to threat but may trigger cardiac events or lead to sudden death [46]. 

If a person is exposed to chronic stress, the stress systems are over-activated and can eventually collapse. Prolonged stress may influence health via several different pathways, i.e., alterations in the autonomic nervous system (increased SNS and decreased PNS activity), neuroendocrine activity and immune, behavioral and cognitive functions [43].

Chronic stress can lead not only to cardiovascular complications such as hypertension, increased risk of angina, myocardial infarction, ventricular arrhythmia and acute heart failure [47] but also to endothelial dysfunction [48], metabolic disorders such as ulcers, irritable bowel syndrome, high cholesterol and triglyceride levels, insulin resistance and diabetes mellitus [49]. Chronic stress induces atrophy in the PFC, which is correlated with working memory, behavioral flexibility and reappraisal impairment [50]. It also decreases dopamine in the brain pleasure circuits, depletes norepinephrine from the LC and reduces frontal lobe serotonin receptor levels, thus contributing to flatness of emotion, concentration weakening, mood dysfunction and bad quality of sleep.

The PNS has received less attention than the HPA axis and the SNS as a relevant stress system because it is not activated but rather downregulated in response to stress [51].

Normally, the SNS and PNS’s activities are in dynamic balance, thus indicating a healthy and flexible physiological system. The autonomic imbalance described by increased SNS and suppressed PNS activity is associated with cellular stress, including an increase in cytokine and reactive oxygen species levels [52].

Stimulation of the SNS fibers descending into the lymphoid tissues can activate macrophages resulting in increased cytokine production. Activity in vagal fibers induces an increase in the transmission of cholinergic ligands into the cholinergic receptors on macrophages, which is mediated by the splenic nerve, resulting in a reduction in cytokine production [53].

Stressfully chronic conditions increase the adrenergic stimulation of macrophages, which increases the production and release of cytokines [54] that trigger the nuclear factor kappa light chain (NF-kB) pathway in activated B-cells [55]. This pathway can cause an increase in of reactive oxygen species that are involved in oxidative telomere damage [56,57]. Acute mental stress in healthy men decreases the vagal tone and delays the recovery of TNF-a, diastolic blood pressure and cortisol for up to an hour after the stressor action stops [58]. In healthy women it was reported that during a speech stressor task, low vagal tone evaluated during HRV was associated with a greater increase in TNF-α and IL-6 but not CRP [54].

However, chronic stress causes hyperactivity of the sympathetic nervous system, which occurs with reduced activation of the HPA axis. Straub et al. termed this physiological phenomenon “uncoupling of SNS and HPA-axis” [59]. In addition to this phenomenon, there is also a reduction in the activity of the parasympathetic nervous system, all of which favor the appearance of chronic diseases.

### 2.3. ANS and the Immune System

Inflammation plays a key role in promoting most chronic diseases such as atherosclerosis, type II diabetes, neoplasia and cardiovascular, respiratory, digestive, neuroendocrine and neurodegenerative diseases. The inflammation can be acute or can progress to a chronic condition. The latter can degenerate into severe forms, leading to cell destruction, organ dysfunction and ultimately death [60].

The immune cells express pattern recognition receptors (PRRs) on their surfaces, which interact with either pathogen-associated molecular patterns (PAMPs) or damage-associated molecular patterns (DAMPs). Activated PRRs and DAMPs oligomerize and assemble different factors, such as nuclear factor kappa B (NF-kB), activator protein 1 (AP1), cellular transcription factor (CREB), CCAAT-enhancer binding proteins (c/EBP) and interferon regulatory factor (IRF) transcription factors, which lead to an increase in the expression of pro- and anti-inflammatory genes [61,62]. The expression of genes encoding cytokines, chemokines and adhesion molecules promotes the further recruitment and activation of leukocytes from the site of the inflammatory process in an attempt to eliminate foreign particles and host debris [60,61]. Cell adhesion molecules and chemokines facilitate leukocyte extravasation from the circulation to the affected site [61].

The adaptive immune system provides highly specific responses to the pathogen. B lymphocytes are involved in adaptive immunity. Additionally, antigen-presenting cells (APCs) provide a link between innate and adaptive immunity. T-cells during maturation can acquire surface markers such as CD4+ and CD8+. CD4+ or T-helper cells, produce cytokines that modulate local immune responses and stimulate CD8+ to produce antibodies. CD8+ also produce cytokines, which promote the destruction of adjacent cells.

APCs uptake foreign proteins and process them in small peptides that are presented to the major histocompatibility complexes (MHC). Dendritic cells, which present MHC class II, activate TH1 or TH2 subtype CD4+ lymphocytes. TH1-cells produce IFN-γ, IL-6, IL-2, IL-12 and TNF-α and are involved in inflammatory processes. Additionally, TH2-cells produce IL-4, IL-10 and IL-13.

The stimulation of the immune cells leads to the release of pro- and anti-inflammatory cytokines, the balance of which is ensured by the ANS, giving adequate host defense with minimal tissue damage.

The HPA axis, the SNS and more recently, the PNS have been shown to regulate the immune system. The HPA axis and the PNS have anti-inflammatory effects and the SNS has been shown to have both pro- and anti-inflammatory effects [62].

A connection is established between the nervous system and the immune system. Peripheral cytokines can signal to the brain through (1) the circulatory system via the active transport of cytokines through the circumventricular organs into the brain [63]; (2) afferent fibers of the vagal nerve, interleukine-1 (IL-1) receptors located in the parasympathetic ganglia in particular [64]; (3) a cellular route whereby chemokines released by activated microglia can attract activated peripheral cell types, including monocytes and T-cells [65]. Signals from cytokines can result in the central and peripheral activation of the SNS.

Cytokines, important mediators of inflammation, are synthesized and secreted by different cells like macrophages, monocytes, lymphocytes, CNS neurons, microglia, astrocytes, oligodendroglia and endothelial cells [64]. Macrophages are implicated in the production of interleukins (IL) 1, 6 and 8 and tumor necrosis factor alpha (TNF-α) and neutrophil produces IL-1b, IL-6, TNF-α and IL-8.

IL-1 and IL-2 activate B- and T-cells, and IL-2 also contributes as a stimulant of B- and T-cell growth and maturation. IL-1 stimulates the production of enzymes that promote the synthesis of leukotriene, prostaglandins and nitric oxide (NO) [66]. Prostaglandin synthesis is induced by the up-regulation of the gene expression of inducible cyclooxygenase type 2 [66]. Cyclooxygenase induces the activation of the HPA axis, which stimulates ACTH and reduces the inflammatory process via corticosteroid release [66].

IL-6 displays both pro- and anti-inflammatory effects, notably mediating the acute phase response to infection. IL-6 enhances ACTH production by activating the HPA axis, and the production of glucocorticoids can potentiate the effects of IL-6.

IL-6 mRNA and IL-6 receptor mRNA have been reported to be localized in several medial hypothalamic areas, including the dorsomedial, ventromedial, and medial preoptic nuclei [67].

Tumor necrosis factor-alpha (TNF-α) can cross the BBB when produced by macrophages in response to insult or injury. In the CNS, it acts as a pro-inflammatory cytokine and can amplify and prolong inflammatory responses through the activation of other cells that release cytokines like IL-1 [64]. TNF induces the gene expression of growth factors, cytokines, cell-surface proteins and acute phase proteins [68]. TNF induces the surface expression of various adhesion molecules, such as intercellular adhesion molecule-1 (ICAM-1), vascular cell adhesion molecule-1 (VCAM-1) and E-selectin, thus promoting the margination of leucocytes at the site of inflammation [68].

Interferons (IFN) are an important mediator of both the innate and adaptive immune responses. Interferons Type 3 are primarily antiviral agents. IFN is a potent activator of macrophages and is responsible for inducing non-specific, cell-mediated mechanisms of host defense [69]. IFN upregulates the expression of ICAMs on activated macrophages, thereby contributing to cellular extravasation [70]. It is released by T-helper 1 (TH 1) and is also a potent inhibitor of the T-helper 2 (TH2) humoral immune pathway. Astrocytes, microglia, and neurons from the CNS express IFNα upon direct viral stimulation, but the major source of IFNα comes from the peripheral dendritic cells.

During inflammation, immune cells recruited at the site of inflammation or cells from affected tissues release chemical attractants (chemokine) that cause leucocytes to adhere to the endothelium and to migrate into the tissue spaces [71]. These chemokines play an important role in responses to disease and heavy exercise [72].

In inflammation, after the release of chemokines from affected cells the attraction of immune cells to the site of injury generates a phase of destruction of the affected tissues mediated by the infiltrating cells. At the same time the body tries to promote tissue repair by producing anti-inflammatory cytokines (IL-4, IL-10, IL-13 and perhaps IL-6), which attenuates inflammation by restricting inflammatory cytokine production and suppressing inflammatory cell activity [73].

IL-4 facilitates humoral immunity, inhibits TH1-cells, and decreases antibody-dependent cell mediated cytotoxicity [73]. Additionally, it decreases the synthesis of IL-1 and reduces the production of NO and ROS [74]. These reductions in ROS and NOS are also found in IL-6 and IL-13 [74].

IL-10, an anti-inflammatory cytokine, in the central nervous system decreases TNF-α, IL- 1β and prostaglandin E2 in the PVN [64]. IL-10 is produced mainly by TH2-cells but also by macrophages, mast cells and B-cells [73]. IL-10 decreases macrophages’ NO production and inhibits the production of IFN by T-cells, thereby delaying the development of cell-mediated inflammation [75]. IL-10 levels are important in the management of inflammatory processes and the destruction of pathogens. When a high level of inflammation is induced, the quantitative level of IL-10 increases to limit the immune response with the purpose of avoiding damaging the host. When IL-10 production is insufficient, the level of pro-inflammatory cytokines increases, leading to damage to the host.

Both primary and secondary lymphoid organs are innervated by the SNS [76]. When the distribution and density of sympathetic nerves in lymphoid organs are not stable there are different changes during immune response [76]. This communication between the SNS and immune cells occurs via the release of NE and subsequent intercellular signaling via postsynaptic adrenergic receptors (ARs) expressed by T-and B-cells, stromal cells, granulocytes, macrophages and mast cells.

During chronic inflammation, the SNS and HPA axis activity are increased, which leads to a local repulsion of SNS fibers from inflamed tissue, including lymphoid organs, to create zones of permitted inflammation [77]. In this phase, there is immune suppression due to the desensitization of glucocorticoid receptors [77]. If this condition is prolonged, overactivation of the SNS can lead to dangerous effects such as toxic shock, tissue damage, immune deficiency and autoimmunity [76].

Stimulation of the β2AR on human T-and B-cells increases cAMP levels and adenylate cyclase activity, suggesting increased gene expression [77]. Under catecholamine and cortisol action, TH2 immunity may be enhanced and the TH1 to TH2 shift will not allow adequate tumor cell surveillance by the immune system [78]. Tumor progression is favored by reduced TH1, impaired antigen presentation, increased T-regs and stimulated angiogenesis [79,80].

Some studies conducted in this field have shown that using β2AR-selective agonists on CD4+T-cell populations either inhibits or enhances the level of interleukin 2, interleukin 4 and IFN-γ, and other studies that used populations of TH1- and TH2-cells obtain results that suggested this type of agent can lower IFN-γ levels and increase IL-4 levels [81]. The TH-cell-dependent IgM antibody levels were assessed in an attempt to study NE depletion in the primary T-cell-dependent antibody response in vivo, and the results show that the levels of the TH-cell-dependent IgM antibody can either decrease or increase [81]. Studies conducted on mice shown an increase in immune cell activity when NE depletion was observed. Other effects observed after antigen exposure were a decrease in serum IgG1, germinal center formation and CD86 expression in B-cells [81]. The fact that the mice that were NE-depleted, unlike the mice in the control group, could not upregulate the expression of CD86 in B-cells implied that NE could be implicated in the regulation of CD86 as a way to increase antibody response [81]. The level of CD86 and IgG1 produced by a B-cell will directly increase if B-cell β2AR stimulation is simultaneously activated or if ab-413 detects T-cells [81]. β2AR stimulation also has an impact on the levels of IgE—studies have found that it increases the level of IgE [82]. The result is that NE has a positive impact on antibody response.

SNS fibers from the superior mesenteric celiac ganglion form the splenic nerve. Preganglionic cholinergic sympathetic neurons innervate postganglionic neurons and these nerves reach the spleen alongside blood vessels, mainly innervating the white pulp and slightly innervating the red pulp [82]. They have also been found at the level of B-cells. This pathway has been named the inflammatory reflex and is controlled by NE and cholinergic neuronal inputs, which result in the attenuated activation of splenic macrophages [83].

In rat model of intravenous endotoxin administration, the bilateral section of splenic sympathetic nerves, increased inflammatory cytokine release; however, bilateral vagotomy was ineffective, which suggests a splanchnic sympathetic efferent reflex arc of the anti-inflammatory neural pathway [84].

The complex interaction between the SNS and the immune system was successfully demonstrated by Straub in 2004. During the electrical stimulation of spleen slices, the release of NE inhibited the secretion of IL-6 via α2ARs under bacteria-free experimental conditions, whereas NE induced the inhibition of IL-6 secretion, which was mediated via βARs under experimental conditions in which bacteria were present [85]. This physiological phenomenon has been called the α-to-β adrenergic switch of NE-induced inhibition of IL-6 secretion [85]. It has also been shown experimentally that TNF-α secretion influences IL-6 secretion, which is more pronounced under experimental conditions where bacteria are present. Previous secretion of TNF-α is important for the α-to-β adrenergic switch of the inhibition of IL-6 secretion by the SNS when conditions are changed from a bacteria-free medium to a medium containing bacteria [85].

NE and E also stimulate vascular endothelial growth factor (VEGF) synthesis [86], matrix metalloproteinase (MMP)-2 and MMP-9 [87] and other pro-angiogenic factors such as IL-6, IL-8, TGF-α, TGF-β and TNF-α [88].

The vagus nerve comprises both sensory afferent neurons and motor efferent neurons, which integrate the information that is delivered to the CNS and control the peripheral effectors from all systems.

Cytokines released by activated macrophages and other immune cells stimulate the sensory afferent vagus nerve fibers, revealing their pro-inflammatory properties [84]. However, a potent anti-inflammatory effect is exhibited by the efferent branch [84].

Despite the fact that there is no neuroanatomical evidence of PNS innervation of the immune organs, there is evidence that the spleen receives both sympathetic and parasympathetic signals [89]. TNF-α secretion by splenic macrophages is inhibited by the vagus nerve via the catecholaminergic fibers from the celiac-superior mesenteric plexus projecting into the splenic nerve [53].

The vagal immune reflex system sends signals to the SNS and the HPA axis centrally, resulting in the peripheral release of anti-inflammatory glucocorticoids and NE [90]; acetylcholine is also released from efferent vagal nerve fibers and results in the negative feedback control of inflammation [64]. The cholinergic anti-inflammatory pathway is mediated by the α7 subunit of the nicotinic receptors (nAChR), expressed on macrophages, monocytes and dendritic cells inhibiting the release of pro-inflammatory mediators such as Il-1β, TNF-α and IL-6 without affecting anti-inflammatory cytokines such as IL-10 [91].

α7nAChRs are expressed in the hippocampus and cortex and have been found in autonomic and sensory ganglia [92]. They have also been found in epithelial and endothelial cells, keratinocytes, lung fibroblasts and leukocytes [93]. α7nAChRs play key roles in proliferation, differentiation, migration, adhesion, cell contact, apoptosis, angiogenesis and tumor progression [93].

In microglial cells, agonist stimulation of α7nAChR inhibits endotoxin-induced TNFα release by reducing p44/42 and p38 mitogen-activated protein kinase (MAPK) phosphorylation [94]. Recently, it was discovered that ACh is produced by T-cells that are regulated by the vagus nerve [95]. Additionally, stimulation of the α7 subunit of the nAChR on neutrophils inhibits their recruitment and the secretion of TNF-α. The removal of the receptor increases TNF-α levels [96].

α7nAChRs play key roles in mediating anti-inflammatory signaling by inhibiting NF-κB and activating the JAK2/STAT3 pathway and are also important for connecting PNS with the sympathetic splenic nerve at the mesenteric ganglion [97]. Likewise, activating the JAK2/STAT3 pathway can lead to the transactivation of NF-κB, which in turn increases the expression of the anti-apoptotic protein Bcl-2 in PC12 cells [98]. Decreased expression and function of α7nAChRs have been associated with neurodegenerative diseases, such as AD, PD and schizophrenia. All these diseases are associated with an inflammatory state caused by hyper-activation of the microglia [99,100].

It was recently demonstrated that increased α7nAChR expression on peripheral blood mononuclear cells was associated with inflammation control, disease severity and clinical outcome in septic patients [101].

Electrical stimulation of the efferent vagal nerve before and shortly after endotoxin administration decreased TNF-α serum levels; however, vagotomy without electrical stimulation increased TNF-α levels [102]. Other studies show that vagal nerve stimulation (VNS) significantly attenuates TNF-α synthesis and improves clinical outcomes in ischemia–reperfusion injury [103].

In summary, both the afferent and efferent vagus nerves mediate anti-inflammatory effects. Afferent vagus pathways are involved in the activation of the HPA axis and adrenal gland corticosteroid release. By contrast, efferent vagus nerves mediate anti-inflammatory processes via a direct effect on immune cells or through the splenic sympathetic nerve. Cytokines released in peripheral tissues activate vagal afferents, resulting in an inflammatory reflex in which efferent vagus nerves inhibit inflammation by suppressing cytokine production via the cholinergic anti-inflammatory pathway.

### 2.4. ANS and Oxidative Stress

Oxidative stress (OS) is defined by the high production or low inactivation of reactive oxygen species and an imbalance between the levels of oxidants and anti-oxidants, with an increased level of oxidants having a destructive and pathogenic effect [104]. Nitrogen species and reactive oxygen (RNS and ROS), when produced at low concentrations, are important for gene expression, cellular growth, infection defense, regulation of cell signaling pathways, regulation of blood flow and control of superior nerve activity.

Excessive amounts of ROS and RNS can be harmful because they can produce lipid peroxidation, proteins and ADN oxidation [105].

The brain is vulnerable to oxidative stress because at this level the concentrations of polyunsaturated fatty acids are high, the catalytic activity is reduced and the antioxidant capacity is minimal [106]. The hippocampus, amygdala and prefrontal cortex are the most vulnerable structures to oxidative stress and consequently the functional decline of these structures is the most prevalent. [107]. Oxidative stress in the brain compromises biochemical integrity of the hippocampus, the amygdala and PFC affecting neuroplasticity and neurogenesis and disturbing normal synaptic neurotransmission as well as neurogenesis factors like brain-derived neurotropic factor.

ROS significantly affect axonal transport and cause the release of pro-apoptotic proteins (such as cytochrome C), which will lead to an increase in blood–brain barrier permeability, neuronal inflammation, impaired synaptic signaling and neuronal apoptosis [108]. In return, the massive apoptosis of neurons can be considered aa potential mechanism of autonomic stimulation attenuation [108,109]. In fact, ROS play an important role in modulating autonomic balance.

Stress responses in neurons result in the inhibition of the neuronal NO synthase (NOS) activity and as a result a reduction in NO production. NOS are present under three isoforms: NOS-1 (in nervous tissue), NOS-2 inducible enzyme (expressed primarily in macrophages) and NOS-3 (in endothelial cells). NO has vasodilator, anti-inflammatory and anti-oxidant functions and acts as a sympatho-inhibitory substance within the central nervous system [110].

NO released from the endothelium inhibits central and peripheral SNS activity and increases central and peripheral PNS activity [111]. This suggests that NO released from endothelial cells may play a role in the modulation of the balance between the SNS and the PNS branches of the ANS. Additionally, NO inhibits the oxidation of LDL-cholesterol, the proliferation and migration of smooth muscle cells, the adhesion and aggregation of platelets and the production of vasodilatation. Inflammation has been shown to downregulate NOS activity. TNF-α has been demonstrated to attenuate NO production by destabilizing eNOS mRNA, which reduces NOS protein expression. The inhibition of TNF restores endothelial-dependent vasodilation in humans [111,112].

High amounts of ROS can be generated in the case of hyperglycemia because it can cause protein kinase C (PKC) activation, an increase in the polyol pathway flux, an increase of the hexosamine pathway flux and an increase in advanced glycation end-products (AGEs) [113]. The increased AGEs will determine an endothelial dysfunction because AGEs bind to the AGEs receptors (RAGEs) on endothelial cells and growth factor and cytokine production is stimulated [113]. ROS production is stimulated by free fatty acids and high glucose levels and this has an impact on NO production, which will be reduced or the bioavailability of nitric oxide will be affected.

Besides NO and ROS, other factors influence the SNS. Some of these factors are implicated in the regulation of the vascular function: endothelin (ET) and the renin–angiotensin system [114]. Normal endothelin levels may suppress SNS activity, whereas endothelin excess may enhance the central and peripheral SNS and influence hemodynamic regulation by the baroreflex, chemoreflexes and vascular tone [114]. Additionally, SNS stimulation can increase endothelin release. Exaggerated SNS activity may impair endothelial function and enhance endothelium-mediated atherosclerosis. On the other hand, since blood vessels provide nutrients for neurons and synapses, endothelial dysfunction has a major impact on the autonomic nervous system. ANS dysfunctions can contribute to endothelial dysfunction, and this leads to worsening ANS dysfunctions, creating a vicious circle that aggravates endothelial functions and impairs angiogenesis.

The renin–angiotensin aldosterone system (RAAS) through angiotensin II (AngII) can stimulate NAD(P)H oxidase and is involved in the generation of ROS. Many studies show that a key mechanism by which AngII influences autonomic dysfunction is via its ability to produce ROS. Ang II produce vasoconstriction and decrease the baroreflex function, sodium and water reabsorption, inflammation and release of aldosterone, vasopressin and noradrenaline. Angiotensin II increases SNS activity and decreases parasympathetic drive [115]. Angiotensin II reduces NO and increase endothelin production. IL-1b, IL-6 and TNF-α are able to stimulate renin and noradrenaline.

High levels of ROS can damage the molecules of lipids, which become precursors of lipid oxidation end-products. Lipid peroxidation can produce changes in the permeability of membranes and can damage enzymatic equipment [116]. Additionally, cytotoxic products resulting from lipid peroxidation contribute to endothelial damage, platelet aggregation, the release of growth factors that stimulate the proliferation of smooth muscle cells and inflammatory response. ROS production stimulates the release of IL-1, IL-6, leptin and adiponectin by monocytes and macrophages through the activation of the transcription factor nuclear kB [116]. Cytotoxic products may also increase the release of chemotactic factor for neutrophils and alter phospholipase A2 activity with the subsequent formation of prostaglandin and end peroxides [116].

**Figure 1:**
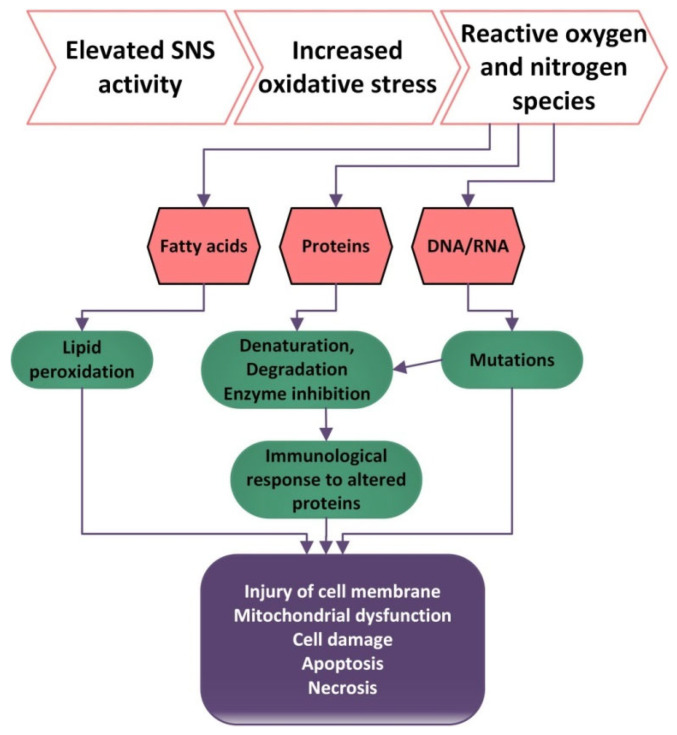
The pathophysiological mechanism by which oxidative stress contributes to the occurrence of various diseases.

Oxidative damage to proteins produces changes in their aggregation and enzyme activity and can also produce proteolysis [106]. ROS contribute to the activation of tyrosine kinases, protein kinase C, and the MAP kinase cascade, which determine the impairment of the cellular responses such as activation, proliferation and differentiation. Additionally, high levels of ROS can cause DNA damage and hence higher frequencies of mutation (Figure 1). The damage to DNA can be countered by DNA repairing processes [117].

Telomeres, which protect chromosomal ends from degradation, are repaired less efficiently than the rest of the genome [118]. Telomeres interact with telomerase, a ribonucleoprotein complex that further influences chromosome-end integrity by adding telomeric repeats to the chromosome 30 end [119]. Telomerase regulates NF-kB-dependent gene expression and NF-kB transcriptionally regulates telomerase levels [119]. It is known that ROS inhibit telomerase activity, generating telomere attrition [120].

Telomere length is associated with psychological and oxidative stress [121]. Greater SNS activation and PNS withdrawal after exposure to physical and psychosocial stressors has been shown to be associated with shorter telomere length in children [122]. PNS withdrawal was measured during HRV by measuring HF. A very interesting study showed that elderly people with shorter telomeres had lower vagally mediated HRV compared to people of the same age group with longer telomeres [57]. Telomerase activity was related to lower vagal tone and greater sympathetic reactivity to an acute mental stressor [57]. HRV is also inversely related to IL-6 and other inflammatory markers, including C-reactive protein. [123]. These discovered aspects indicate that low vagal tone correlates with increased amounts of cytokine-induced activation of NFkB and, in turn, with increased ROS production. Thus, the reduction in PNS activity stimulates the inflammatory process and the production of ROS, which leads to reduced telomere length [123].

Antioxidant enzymes such as superoxide dismutase (SOD), glutathione peroxidase (GPx), catalase (CAT) and other antioxidant molecules are the defense used against ROS: ascorbic acid (vitamin C), tocopherol (vitamin E), vitamin A, flavonoid and ubiquinone. In response to ROS, cells increase their antioxidant defenses through the activation of nuclear factor erythroid 2–related factor (Nrf2), which increases the expression of several endogenous antioxidants [124].

Recent studies have revealed a close connection between oxidative stress and inflammation, with each of these two processes influencing the other and creating a vicious circle capable of generating and maintaining an inflammatory process.

### 2.5. Physical Exercise

Regular physical exercise is a key factor for the prevention of many chronic diseases [125]. Physical exercise (PE) can be used as a primary non-pharmacological clinical tool because it can improve antioxidant capacity, reduce oxidative stress and inflammation and increase energy efficiency. Depending on the volume, the intensity and the frequency of exercise, acute or chronic biochemical and physiological responses are induced. PE intensity is usually expressed as a percentage of the individual’s maximum oxygen uptake (VO2max), which is the maximum aerobic capacity during PE. Metabolic equivalent (MET) is defined as the amount of oxygen consumed at rest, and it is an optimal method used to describe the functional capacity or tolerance of an individual to certain PEs [126,127]. One MET is equal to 3.5 mL/kg/min oxygen uptake. Using VO2max and MET, exercise can be classified as light (VO2max <37–45%, MET <2–2.9), moderate (VO2max 46–63%, MET < 3–5.9), vigorous/intense (VO2max 64–90%, MET <6–8.7) or maximal/dangerous exercises (VO2max ≥91%, MET ≥8.8) [127].

According to the type of contraction, effort can be divided in isotonic (dynamic), isometric (static) and isokinetic. Based on the oxygen supply of the body, there are aerobic, anaerobic and mixed effort types.

The positive impact of physical activity is well known and has been studied by many researchers. But physical activity may also have negative impacts on the body, depending on the type of effort, the duration of the effort, and the individual characteristics of the person exerting the effort (age, gender, diseases, etc.). These negative impacts are less well studied and seem to be linked with the oxidative stress and inflammation induced by effort, mainly reflected in the increase in oxidants and decrease in antioxidants during physical activity. Due to the fact that the level of antioxidants in the body decreases with age, age is an important factor in the body’s response to oxidative stress.

### 2.6. Physical Exercise and Oxidative Stress

During exercise, an increase in respiration and oxygen uptake directed to the body’s vital organs take place. Increased oxygen consumption due to higher energy requirements results in increased levels of reactive oxygen and nitrogen species [128]. ROS and other free radicals produced cause oxidative stress at the level of vital organs and this causes cells to defend themselves using antioxidants. Antioxidants can be divided into endogenous antioxidants (glutathione; vitamins C, A and E; uric acid; and iron binding protein) and antioxidant enzymes (AOE) (superoxidase dismutase, CAT and glutathione peroxidase). AOE activity undergoes changes due to modifications in the consumption of oxygen in the body (oxidative stress). The systemic levels of antioxidant during exercise depend on the type, mode, intensity, frequency and duration of the exercise. During exercise, the blood flow is increased to the vital organs and muscles but is lowered to the liver, and this has an impact on antioxidant levels. The intra and extracellular transportation of glutathione is affected and the synthesis and degradation of glutathione is also affected. This explains why the efficacy of antioxidant systems differs after acute exercise and exercise training [129].

Over the years, studies conducted on the impact of PE on the body in general, but also on elderly people in particular, have shown a positive impact of PE on lowering the risk of age-related diseases. PE can impact the activity of antioxidants during effort, and this is one of the mechanisms considered to be implicated in lowering the risk of age-related diseases. Studies have also been published showing a connection between the intensity of the effort exerted and oxidant and antioxidant levels. Oxidative stress seems to reach higher levels during high-intensity acute exercises. This connection was studied by Vezzoli et al., with the aim of assessing the impact of high-intensity discontinuous training (HIDT) on oxidative stress and damage. In the study, 20 long-distance master runners were asked to participate in an eight-week training program. Ten of the runners were included in continuous moderate-intensity training and ten of them were included in high-intensity discontinuous training. Oxidative damage markers (thiobarbituric acid reactive substances, protein carbonyls, 8-hydroxy-2-deoxy-guanosine and total antioxidant capacity) were used to assess the participants before and after the training. There was no difference between the two groups regarding the levels of oxidative stress induced by exercise and the beneficial effects of training on redox homeostasis were similar [130]. The nitric oxide/redox-based signaling is increasing during intermittent high-intensity effort and this may explain the results of the previous study. High signaling could have an impact on sympathetic outflow and endothelium-dependent relaxation in relation to the increased expression of the genes implicated. High levels of ROS caused by effort induce the activation of antioxidant defenses and this causes a positive adaptation of both the CNS and PNS [130].

Yen et al. studied the impact of exercise training on a group of 42 patients undergoing chemotherapy for head and neck cancer because it has previously been shown that chemotherapy has a negative impact on fitness performance and can cause an increase oxidative stress. The patients were included in an eight-week exercise course that included aerobic and resistance exercises carried out three days a week. Blood pressure and heart rate were used to assess the exercise capacity and responses, showing an increase in the exercise capacity and an amelioration of exercise responses. Blood pressure at rest was decreased, with an increase 1 to 3 min after the physical exercise. Oxidative stress markers (8-hydroxy-20-deoxyguanosine, malondialdehyde, and carbonyl content) were also evaluated, along with total antioxidant capacity. The levels of oxidative stress were decreased, and the levels of antioxidants were increased. The results of the study show that in this category of patients training can decrease systemic oxidative stress and it also has a positive impact on exercise capacity and response [131].

Moderate physical activity has a positive impact on the body because it helps maintain the health of bones, muscles and joints; helps maintain normal levels of cholesterol and body weight; and also decreases levels of cholesterol and overweight. During this type of effort, the level of free radicals produced is moderate and the body can adapt. The body also tries to adapt during exhaustive physical activity but the levels of oxidants produced are much higher so this will cause an imbalance between oxidants and antioxidants resulting in oxidative damage (lipid oxidation, protein oxidation and DNA oxidation). This makes body more vulnerable to fatigue, injury and disease [132].

In a study conducted by González-Bartholin et al., ten older healthy subjects were asked to perform 30 min work-outs that included different types of exercises (moderate-intensity concentric and eccentric cycling and high-intensity eccentric cycling) in a randomized manner. These exercises included moderate-intensity concentric cycling with 50% maximum power output, moderate-intensity eccentric cycling with 50% maximum power output and high-intensity eccentric cycling with 100% maximum power output. After the exercises were conducted, the effects of different types of exercises were studied by measuring VO2 and HR and the results showed that high-intensity eccentric cycling had a greater impact on VO2 and HR. The next day, the subjects were examined again and the researchers looked at the muscle strength loss, peak soreness, creatine kinase activity, malondialdehyde levels and IL-6 levels. Muscle strength loss and peak soreness were greater in subjects that performed high-intensity eccentric cycling and the activity of creatine kinase was high in these subjects, along with IL-6 levels. MDA levels did not decrease after any type of exercise. This study, even though it was conducted on a small number of participants, shows a connection between the intensity of the effort and the impact of oxidative stress [133].

Dantas des Lucas et al. conducted a study in this field that included 11 subjects to assess the impact that ultra-endurance exercise have on platelet oxidative metabolism, blood oxidative stress markers and neopterin levels. The subjects were well-trained male athletes who agreed to participate in the study and to participate in a race (90 km of alternating off-road running, kayaking and mountain biking). Blood samples were collected 12 h before the race and 15 min after the race. The parameters measured were: lactate dehydrogenase, creatine kinase, respiratory chain complexes I, II and IV activities, lipid peroxidation, catalase, protein carbonylation, oxygen consumption and neopterin levels. Fifteen minutes after the race, all parameters were much higher than 12 h before the race, which shows that after intense physical activity oxidative stress, muscle damage and immune system activation are increased [134].

The effects of professional training regarding redox balance were studied by Tong et al. In this study, 10 adolescent runners were included and the effects of a 21 km running time trial on the status of oxidants and antioxidants were evaluated twice in a year. The serum concentrations of thiobarbituric acid-reactive substances (TBARS), reduced glutathione (GSH), xanthine oxidase (XO), superoxide dismutase (SOD), catalase (CAT) and total antioxidant capacity (T-AOC) were determined before and 4 h after the 21 km run. The serum concentrations of TBARS and SOD were lower after the run, while XO, CAT, TAOC and GSH remained the same as before. At the subsequent evaluation the levels of TBARS and SOD were lower and XO and CAT levels were higher post-exercise. The results seem to show that professional training in this category of individuals does not interfere with the evolution of their antioxidant defense [135].

The autonomic nervous system seems to play an important role in the way an organism reacts to oxidative stress because it is related to a decrease in oxidative stress induced by physical effort. The divisions of the autonomic nervous systems include the internal organs, skin and muscles and controls their function by producing and secreting acetylcholine, adrenaline and noradrenaline. In this way, ANS is capable of influencing the response of the body to stress and inflammation [136]. The adaptation of the ANS is one of the ways in which the positive impact of exercise is achieved. The recommendations regarding moderate-intensity exercises for most people are 30 min/day 5 days/week. For people with diseases such as autonomic disorders, training should be carried out under expert supervision [137].

Exercises conducted on a daily basis can cause the ANS to adapt to parasympathetic dominance, which translates to a lower HR at rest. Nitric oxide seems to be associated with bradycardia induced by exercise and studies have shown that the transfer of nitric oxide synthase into the atrial wall has the same effect as the exercise-induced vagal phenotype. HRV and muscle sympathetic nerve activity (MSNA) are useful, objective and reliable methods used to assess the autonomic nervous system’s activity. HRV can be easily assessed with the help of an electrocardiograph. MSNA is used to directly determine the sympathetic nerve activity at the level of the peroneal nerve using microneurography and this is considered the “gold standard” when assessing the intensity of sympathetic outflow. In a review, A.J. Hautala et al. showed that regular aerobic fitness training can cause an increased cardiac vagal modulation of the heart rate and they also showed that normal or pathological functioning of the ANS causes individual responses to aerobic training. Individuals with high vagal modulation at the start of the training obtain greater improvements in their VO2 peak. The use of methods to assess and monitor the ANS can help optimize the exercises chosen for aerobic fitness [138].

Because studies show that in patients with obesity the activity of the sympathetic nervous system and oxidative stress are high, Li et al. investigated the impact that exercise had on four groups of rats with different diets. One of the groups received a high-fat diet for 12 weeks. Rats from the group with a high-fat diet and the ones from the group that received a regular diet were trained on a treadmill 5 days/week 60 min/day for eight weeks. The activity of the sympathetic nervous system was assessed by measuring the plasmatic levels of norepinephrine and oxidative stress was assessed by measuring the plasmatic and muscular levels of malondialdehyde, superoxide anion and F2-isoprostanes. The results showed that in the group of rats who underwent exercise training the activity of the sympathetic nervous system and oxidative stress was lower compared to the activity in the three other groups of rats [139].

Menopause has been identified as a risk for cardio-metabolic disorders and combined training (resistance exercises and aerobic exercises) seem to have a positive impact on this type of disorder. Conti et al. conducted a study with the purpose of evaluating the impact of combined training on blood pressure. Inflammation and oxidative stress were measured in ovariectomized rats suffering from hypertension with fructose overload. The rats included in the study were divided into three groups with different levels of exercise and blood pressure: sedentary but normotensive and sedentary or trained ovariectomized hypertensive rats with fructose overload. The combined training was performed for eight weeks with 40–60% maximal capacity output using treadmills and ladders on alternate days. Blood pressure was determined directly and oxidative stress and inflammation levels were determined using cardiac and renal tissues. The rats included in the third group had increased insulin resistance, cardiac inflammation and oxidative stress parameters. The combined training had a positive impact on atrial pressure, heart rate, sympathetic modulation and insulin resistance. Additionally, nitric oxide bioavailability was higher, TNF-α was reduced, high levels of IL-10 were identified in the cardiac tissue and high levels of antioxidants were present in the cardiac and renal tissue of the rats who underwent training. The conclusions of the study were that risk factors such as menopause can have a negative impact on oxidative stress and metabolic, autonomic, cardiovascular and inflammatory parameters and that combined training has a positive impact and can attenuate this dysfunction [140].

Obesity is another factor that can increase sympathetic activity and oxidative stress. Li et al., studied the role of exercise in decreasing sympathetic activity and oxidative stress in obese rats. The rats included in the study were divided in four groups (regular diet sedentary rats, regular diet exercise training rats, high-fat diet sedentary rats and high-fat diet exercise training rats). The rats included in the second and last group underwent a training program on a treadmill 60 min/day, 5 day/week for eight weeks. The plasma level of norepinephrine was used to assess the activity of the sympathetic nervous system and oxidative stress was assessed by measuring superoxide anion, F2-isoprostanen and MDA serum concentrations. The rats with a high-fat diet presented lower levels of norepinephrine and oxidative stress parameters, which suggests that exercise can attenuate the impact of the sympathetic nervous system and oxidative stress in obesity [141].

Cikrikcioglu et al. performed a study on the oxidative stress and autonomic nervous system function of patients suffering from restless leg syndrome (RLS) because the implication of oxidative stress in this disease has not been previously studied. One hundred patients diagnosed with RLS were included in the study and divided into two groups. Group one included 50 untreated patients with RLS and the second group consisted of 50 healthy controls or controls who suffered from mild iron deficiency. The controls had the same ages and genders as the RLS group. Markers were used to assess oxidant and antioxidant levels and the HRV was also determined. The results showed that total oxidant status and arylesterase and paraoxons levels were increased while lipid hydroperoxides, acetyl cholinesterase and butyryl cholinesterase levels were decreased in the restless leg syndrome group. Regarding HRV, in the first group the HRV triangular index was lower than in the control group. The authors concluded that the high level of acetyl cholinesterase and low levels of lipid hydroperoxides in the RLS group seemed to be related to the effort of the organism to protect the dopaminergic activity in the CNS. The patients in this group showed an increase in sympathetic activity and this might be a method of alleviating RLS symptoms; however, it also causes an increase in total oxidant status [141].

Polii et al. conducted a study on individuals suffering from chronic fatigue syndrome in order to establish whether oxidative stress is connected to pain symptoms and modification of pain after exercise. The authors also wanted to assess the possible existing relationship between the parasympathetic activity of the vagal nerve and oxidative stress by comparing patients diagnosed with myalgia encephalomyelitis or chronic fatigue syndrome and healthy sedentary people. Participants were asked to complete a submaximal exercise test and the cardiorespiratory parameters were continuously monitored during the test. Oxidative stress was assess using the levels of thiobarbituric acid reactive substances, vagal activity was measured using HRV and a visual analogic scale was used to assess the level of pain felt by patients. The level of pain before and after the test was higher for the group that included patients suffering from ME/CFS and pain was decreased after exercise in the healthy group. Oxidative stress levels did not suffer any modification after exercise in either group. Levels of TBARS were correlated with the levels of pain before and after the tests were performed, but only in patients suffering from ME/CFS. Another correlation found was between exercise-induced modification of HRV and TBARS in the healthy group. The conclusions of the authors were that oxidative stress is associated with pain symptoms in patients suffering from ME/CFS and that the parasympathetic activity changes in healthy subjects are partially correlated with oxidative stress changes [142]. 

Because overload training (large volume or long-term exercise) causes oxidative distress, this will nullify the positive impact of the physical training on health outcomes. The kinds of physical exercises recommended due to the increased levels of antioxidant enzymes they generate are moderate exercises that can improve individuals’ physiological and functional capabilities. During this type of exercise, MAP kinases and NF-kappa B pathways are activated [143].

### 2.7. Physical Exercise and Anti-Inflammatory Effects

Skeletal muscle, the largest organ in the body, can produce myokines, firstly in the form of a sequence of pro-inflammatory cytokines (IL-1, IL-6, IL-8, IL-12, TNF-α, IFN-γ, VEGF and IL-1β) and then in the form of regulatory, anti-inflammatory cytokines (e.g., IL-2, IL-4, IL-10, IL-11, IL-13 and IL- 1ra), in response to contraction [144,145]. Myokines may be involved in mediating the health-beneficial effects of exercise and play important roles in protection against diseases associated with low-grade inflammation, insulin resistance and hyperlipidemia such as cardiovascular diseases, type 2 diabetes mellitus and cancer.

Regular PE, if guided correctly, can modulate neurobiological and neuroinflammatory mechanisms to generate anti-inflammatory responses, which are the key factors in improving overall health and controlling the persistent inflammation that is characteristic of chronic diseases.

It has been demonstrated that the plasma concentration of IL-6 increases in an exponential manner during muscular exercise [146]. The peak IL-6 level is reached at the end of the exercise or shortly thereafter [146]. IL-6 plays a fundamental role in the anti-inflammatory process resulting from exercise, and it presents both pro- and anti-inflammatory characteristics [147].

Mathur and Pedersen in 2008 demonstrated that IL-6 myokine and IL-6-induced acute phase proteins cause anti-inflammatory and immunosuppressive effects by lowering the pro-inflammatory response of the immune system [148]. In a recent study, LuzScheffer and Latini showed that regular PE with light / moderate intensity causes an anti-inflammatory response by stimulating the production of IL-6 and neopterin that subtly reduce the risk of infection and chronic non-communicable diseases and generate neuroprotection [149]. IL-6 is the most studied cytokine in relation to PE which, depending on its intensity and volume, can have a pro- and anti-inflammatory effect.

Ostrowski et al. have shown that prolonged running increases levels of IL-6 up to 100 times in the bloodstream, and Pedersen et al. have shown that prolonged running has a positive effect on glucose uptake [150]. When PE is carried out in moderation, IL-6 becomes an anti-inflammatory cytokine that limits the production of IL-1b and TNF-α [151].

Alizaei Yousefabadi et al., in a systematic review of twenty randomized controlled trials (RCTs), concluded that isolated aerobic exercises and aerobics combined with resistance exercises are optimum for reducing the inflammatory markers of metabolic syndrome (MetS). The efficacy of this regimen can be seen using two parameters. The first parameter is the increase in the concentration of IL-10, which shows a mean difference of −0.48 pg/mL. The second parameter is the decrease in the concentration of pro-inflammatory cytokines in the bloodstream: TNF-α has a mean difference of −1.21 pg/mL, IL-8 shows a mean difference of −0.31 pg/mL and CRP has a mean difference of −0.79 pg/mL [152]. 

Balducci et al. conducted a study on 82 patients with MetS and type 2 diabetes mellitus that were randomized into four groups: A—sedentary group, B—intense PA, C—high-intensity aerobics, D—aerobics with resistance training. They investigated the effect of these different PE regimens on high-sensitivity C-reactive protein (hs-CRP) and pro-inflammatory and anti-inflammatory cytokines IL-4 and IL-10. They concluded that in over 12 months of training, hs-CRP decreased significantly only in groups C and D. The secretion of pro-inflammatory IL-1b, IL-6, TNF-α and IFN-γ cytokines and anti-inflammatory IL-4 and IL-10 cytokines was only inhibited in group D [153]. 

Dadrass et al., in a randomized, placebo-controlled, double-blind clinical trial found that patients aged 40–65 with MetS and type 2 diabetes mellitus who underwent 12-week endurance training with vitamin D supplementation had improved serum IL-6, CRP and TNF-α levels. IL-6, CRP and TNF-α serum levels decreased in the PE group, but even more so in the PE group with vitamin D supplementation (5000 IU every two weeks for three months) [154]. 

Starkie et al. hypothesize that exercise-induced IL-6 works to reduce TNF-α production induced by low-grade endotoxemia. To test this hypothesis, eight healthy men participated in three experiments in which they (1) cycled at 75% VO2max for 3 h, (2) were infused with recombinant human IL-6 (rhIL-6) for 3 h while they rested and then (3) rested for another 3 h. After two and a half hours, the subjects received an intravenous bolus 0.06 ng/kg of lipopolysaccharide Escherichia coli endotoxin to generate a low-grade inflammation that caused an increase in plasma TNF-α levels. They concluded that PE increased IL-6 and rhIL-6 and completely attenuated the endotoxin-induced TNF-α. PE and the infusion of rhIL-6 at physiological concentrations inhibit endotoxin-induced TNF-α production in humans, suggesting that myokin IL-6 mediates anti-inflammatory activity [155].

Rosenbaum et al., in an RCT, examined the effects of a 3- to 4-month school intervention on the type-2 diabetes risk (insulin resistance and pro-inflammatory condition) of school-based adolescents (8th grade). They evaluated the effects of (1) health hours, (2) nutrition and exercise and (3) an aerobic exercise program. The following parameters were evaluated: body fat (bioelectric impedance), insulin sensitivity, cell function, lipid concentrations, and circulating levels of IL-6, CRP, and TNF-α. The intervention resulted in a decrease in body fat and circulating levels of CRP and IL-6 and a decrease in insulin resistance regardless of somatotype and intervention schedule. The adolescent lifestyle influences the circulating levels of IL-6 and CRP, while increased PE is associated with high insulin sensitivity. They concluded that nutrition and exercise at school are extremely beneficial and reduce type 2 diabetes risk factors [156].

Salamat et al. studied the response of pre-inflammatory cytokine factors to PE in 43 healthy overweight men (BMI = 28.56 ± 2.67). Their study investigated the concomitant PE performance of different training patterns (endurance, resistance and endurance + resistance (concomitant)) on pre-inflammatory cytokines in overweight young men. The pre-inflammatory cytokines IL-6, IL-1b and TNF-α were measured after eight weeks of training. At the end of the training, the results showed a significant difference between IL-1b (*p* = 0.046) and IL-6 (*p* = 0.009) compared to the initial value, while TNF-α levels remained unchanged. IL-6 showed significant differences (*p* = 0.020) between the endurance and resistance groups. IL-1b and IL-6 showed considerable differences between the endurance and concomitant groups, demonstrating that PE had a positive effect on pre-inflammatory cytokines [157].

Chen et al. have shown that TLR-4 regulates inflammatory reactions after regular aerobic PE training. Long-term aerobic PE can effectively attenuate TLR-4 expression, which also reduces the secretion of cytokines by inflammatory cells, thereby boosting immunity [158].

Pedersen et al., demonstrated that high-intensity, long-term PE (marathon running) suppressed immune function for a period of several hours to days, increasing the risk of infections, as was confirmed by Goh et al. in 2019 [159,160]. Pedersen and Ullum showed that high intensity PE had antagonistic effects compared to moderate PE. The study was conducted on six healthy individuals who did a 25%, 50% and 75% VO2max ergometric bike program for one hour. Blood samples were collected 2 h after the end of the PE. After moderate PE, no immunosuppression was recorded but prolonged high intensity PE caused the down-regulation of the immune function. They suggest that natural killer cells are highly influenced by PE and the mechanisms behind the changes induced by intense exertion are related to cytokines, adrenaline, noradrenaline, cortisol, stress and growth hormones, hyperthermia and beta-endorphins. This causes high-performance athletes to have high levels of natural killer cells at rest, while after acute high intensity PE their levels drop dramatically, leading to immunosuppression and low resistance to pathogens [161].

Cooper et al. showed that high-intensity PE could be dangerous and is associated with chronic musculoskeletal injury, anaphylaxis, and sudden death. High-intensity PE generates a “dangerous” immune stress and inflammatory response that can become harmful to health in certain circumstances. The inflammatory response is characterized by an increase in potent inflammatory mediators’ blood concentrations with the mobilization of an increased number of leukocytes in the central circulation. Natural killer cell, lymphocyte and monocyte levels increase rapidly with the onset of PE, but begin to decline immediately upon cessation. Physical overload is associated with musculoskeletal injuries and delayed-onset muscle soreness that causes systemic increases in TNF-α and IL-1, which indicate inflammation [162].

Tidball demonstrated that the proinflammatory response generated by PE plays a role in the recovery process of damaged muscles and that this response is associated with a complex situation in which inflammatory cells promote both injury and repair through the combined actions of free radicals, growth factors and chemokines. Muscle damage produces an inflammatory response in which neutrophils invade rapidly followed by macrophages, which coincide with muscle repair that involves the activation and proliferation of satellite cells followed by their differentiation. In contrast to other high-intensity PE studies that show the destructive role of high intensity exercise, this study shows that inflammatory cells can promote both injury and repair mechanisms [163].

Suzuki et al., documented the systemic kinetics of cytokines after PE, especially TNF-α and IL-1b, which induce cytokines in acute phase reactions. They found that the circulating concentration of these cytokines remains almost unchanged after exertion. Plasma interferon (IFN)-alpha and IFN-gamma remain unchanged, while IL-2 decreases and IL-8 increases after endurance exercises. They concluded that long-duration high-intensity PE suppresses the production of immunomodulatory cytokines [164,165].

Rokitzki et al., evaluated TNF-α levels immediately following a marathon run and discovered high values of TNF-α [166]. Moldoveanu et al. observed a 90% increase in plasma TNF-α following 3 h of endurance exercise at 60 to 65% of VO2max [167].

From the studies described, it is clear that the intensity, type and duration of exercise and the muscle mass involved in the exercise influence the secretion of cytokines into the circulation. Thus, high-intensity and long-duration PE can be dangerous from the point of view of the secreted inflammatory cytokines [168]. Concentric muscle contractions in contrast to eccentric exercises are associated with higher amounts of plasma IL 6. It was demonstrated that muscle damage is not required to increase plasma IL-6 during exercise [169]. 

Fibroblasts, myoblasts, endothelial cells and smooth muscle cells have been shown to be capable of producing IL-6. Skeletal muscle cells are capable of producing IL-6 in response to reactive oxygen species that are produced as a result of the oxidation of fat and glucose.

A small net release of IL-6 from the internal jugular vein has been reported, suggesting that the CNS may contribute to the IL-6 found in the circulation [170]. In the brain, IL-6 predominantly comes from activated astrocytes [171]. IL-6 levels in the plasma increase rapidly during exercise, whereas the production of IL-6 in the brain increases more slowly [172].

Levels of other cytokines that are expressed in the skeletal muscle following exercise, such as TNF-α and IL-1β, increase, but the circulating concentration of these cytokines does not change (or only increases slightly) [172]. Conversely, the circulating concentrations of IL-1 receptor antagonist (IL-1ra) and IL-10 increase markedly, but these cytokines are not expressed in skeletal muscle after exercise [172].

There are studies that show that physical exercise can alter the inflammatory mode of microglial cells. Microglia, the primary immune cells in the CNS, can be activated by the M1 (pro-inflammatory subtype) and M2 (anti-inflammatory subtype) pathways. The M1 secretes pro-inflammatory cytokines and free radicals that are toxic to the surrounding cells. The M2 secretes anti-inflammatory cytokines and promotes tissue healing by secreting trophic factors.

Sung et al. demonstrated that 30 min of treadmill exercise five days a week at speeds of up to 12 m/min could reduce microglial activation by decreasing the expression of the inflammatory enzyme iNOS. Exercise in mice can increase levels of the growth hormone insulin growth factor 1 (IGF-1) in the prefrontal cortex and hippocampus, which have an anti-inflammatory effect by stimulating the M2 macrophage phenotype [173]. Physical exercise can switch microglial cells from inflammatory M1 to anti-inflammatory M2 types.

Both adrenaline and cortisol rapidly increase during physical training and could be related anti-inflammatory pathways. β2-adrenergic receptor stimulation of microglia inhibits their activation by inhibiting NF-κB [174]. The levels of β2AR on the cell membrane of macrophages are downregulated following exercise in humans [175]. This β2AR downregulation occurs in over-trained subjects but not after moderate exercise. This implies that these receptors still function normally and thus inhibit the expression of pro-inflammatory cytokines such as IL-12 after moderate exercise [176]. Physical exercises can downregulate TNF and TRL4 and allow monocytes to enter an anti-inflammatory mode [177]. The inhibition of this pro-inflammatory response can restore hippocampal neurogenesis [178].

Exercise can lead to increased levels of neurotrophic factors, especially nerve growth factor (NGF), brain-derived neurotrophic factor (BDNF) and insulin-like growth factor (IGF-1). BDNF plays many important roles in neuroplasticity, neuronal growth and differentiation. Physical exercise has been found to normalize BDNF. It has been suggested that higher aerobic fitness levels are associated with larger hippocampal volume and improved neuronal health and that acute aerobic exercise can induce increased BDNF levels in the peripheral blood [179]. Other studies have shown that acute stress and cortisol administration can lead to reduced BDNF levels [180].

Exercise can also increase endorphin levels. β-endorphins are endogenous opioid neuropeptides that play a role in relieving pain and inducing wellbeing. In the brain, β-endorphins are considered neurotransmitters as well as neuromodulators because they are more efficient and stable on more distant targets than other neurotransmitters. They are produced by pro-opiomelanocortin (POMC) cells in the hypothalamus and pituitary gland [181].

Hawkes demonstrated that PE can contribute to the natural production of endorphins. The phenomenon of “wellbeing” in which PE is involved is the consequence of three mechanisms by which endorphins are stimulated: the ‘runner’s high’, addiction to exercise and pain tolerance [182].

Schwarz and Kindermann have shown that sustained PE increases the peripheral concentration of β-endorphins, which is directly related to the perception of pain and the mood of the individual. They analyzed the function of β-endorphins during PE by investigating changes in opioid concentrations in comparison with other stress hormones depending on the exercise intensity and duration. The results of the study showed that peripheral β-endorphin levels during exercise are directly influenced by the effort intensity and duration. In short term aerobic exercise, the degree of metabolic demand is a decisive factor for the release of β-endorphins that is correlated with the concentration of lactate, suggesting that endogenous opioids have a direct influence on acidosis tolerance and anaerobic capacity. The levels of β-endorphins are constant during endurance exercises because a state of equilibrium with lactate is induced and the levels of opioids increase exponentially one hour after exercise [183].

Siswantoyo and Aman demonstrated that breathing exercises affect immunoglobulin G (IgG) levels and glucose secretion in people aged 45–50 years old. Ten patients underwent a seven-week PE program consisting in 21 sessions of regularly performed and programmed exercises that combined breathing, PE and concentration. In this study, blood samples from the ulnar vein were obtained and analyzed with an IgG ELISA and a glucometer. The results showed a significant increase in β-endorphin, with an average increase of 922 mg/mL, and a significant increase in IgG, with a mean rise of 33.226 ng/mL, while blood glucose significantly decreased with an average decrease of 28.9 mg/100 mL. The authors of this study concluded that regular breathing exercises may increase the secretion of β-endorphins and IgG, cause a decrease in blood glucose levels and improve immunity [184].

Dietrich and McDaniel argue that “endorphin theory” is unsustainable because the effects β-endorphins on PE are unclear due to the fact that the “exercise endorphin connections” are ambiguous, leaving room for interpretation [185]. The amino acid sequence of β-endorphin is almost identical to the sequence of the adrenocorticotrophic stress hormone, which increases in PE, leading to serious detection problems. Another inconsistency was found in the physiological and biochemical responses to endurance PE because β-endorphin should bind tightly to the µ opioid receptor (MOR). MOR has an increased affinity for beta-endorphins, being the primary psychoactive alkaloid in opium. The β-endorphins and MOR are part of the endogenous opioid system that mediates the euphoric and analgesic properties of opiates. The activation of this endogenous opioid system, which is responsible for severe respiratory depression, gastrointestinal motility inhibition and pinpoint pupils, is not observed in running athletes. The most serious limitation of “endorphin theory” is the measurement of β-endorphins from the bloodstream, as it cannot be considered an indicator of central effects due to the endorphins being too large to cross the blood–brain barrier [186].

Anandamide (AEA), an endocannabinoid (eCB), is a fatty acid neurotransmitter and is an endogenous binder of the same receptors involved in the effects of cannabis. AEA participates in the body’s endocannabinoid system and mimics many of the pharmacological effects of Δ-9-tetrahydrocannabinol (THC). Just like THC, AEA has two main molecular targets in the form of cannabinoid CB1 and CB2 receptors [186].

Sparling et al. demonstrated that PE activates the endocannabinoid system through a neurohumoral mechanism, resulting in exercise-induced analgesia. The study was conducted on 24 males with an average age of 23 years, BMI 74.5 ± 7.9 kg and height 183.7 ± 6.2 cm divided into three groups. After a 5 min warm-up on the running band and cycle ergometer, the intensity of the exercise was slowly increased to obtain a heart rate in the range of 70–80% of the maximum heart rate of 140–160 bpm. After the desired intensity was reached, the subjects ran and cycled for another 45 min, followed by a 50 min break until blood was collected. The results showed that plasma AEA levels were significantly elevated in runners *p* < 0.01 and cyclists *p* < 0.05 but low in the inactive control group. The study shows that moderate-intensity PE activates the endocannabinoid system, suggesting that this is a mechanism for exercise-induced analgesia [187]. This mechanism is activated by the dramatic increase in bloodstream levels of AEA, suggesting that AEA acts on the peripheral sensory fibers to reduce pain [187].

Stensson et al. confirmed that AEA increases after PE in patients with fibromyalgia, managing to control chronic pain. The aim of the study was to investigate the effects of resistance PE on AEA and related lipid levels in fibromyalgia in a group of 37 women suffering from this disease. The subjects came from three centers, were aged 20–65 years, had been diagnosed with fibromyalgia and underwent a 15-week progressive resistance training scheme. After the 15-week program, plasma AEA levels were increased, indicating that chronic resistance PE has analgesic and pain-control effects [188]. In a systematic review, Barbosa et al. confirmed that resistance training is a non-pharmacological method of treatment that can alleviate the effects of fibromyalgia in adult women [189].

Marin Bosch et al. have shown that elevated AEA levels in PE stimulate hippocampal and striatal activity and function, thereby strengthening memory for motor sequences. Acute PE has the ability to improve memory function by increasing neuronal plasticity in the hippocampus, which encodes representations of episodic memory. The study looked at the impact that acute PE has on motor sequence learning and its underlying neurophysiological mechanisms in humans. The study used a randomized cross-over design on 15 healthy and fit males with an average age of 23.2 years who followed a moderate intensity FET program at 70% maximal heart rate (60% of VO2max), a high-intensity FET at 80% maximal heart rate (75% of VO2max) and one rest condition. Circulating AEA levels, functional MRI activity, and subject behavior were determined. The results showed that PE improved motor sequence memory significantly after high-intensity exercise and acceptably after moderate-intensity exercise, which was correlated with increased circulating levels of AEA and caudate nucleus and hippocampus activity [190]. Thomas et al. substantiated the dose–response relationship in favor of high-intensity PE to strengthen procedural memory. They found that consolidating procedural memory requires a single period of high-intensity aerobic exercise of more than 90% VO2max. The study included 36 healthy male subjects who were randomly divided into three groups who performed different types of exercises. In first group were subjects who performed a single cycle of aerobic exercise at 90% maximum power. A second group with subjects who performed a single cycle of aerobic exercise at 45% maximum power and a third group with subjects who did not undergo any type of exercise 20 min after learning a motor skill. The conclusions of the study were that exercise intensity plays an important role in modulating the effects that a single PE course has on the consolidation phase after learning motor skills [191].

An increasing number of studies support the idea that PE has a positive effect on cognitive function. In a recent randomized controlled study, Farinha et al. observed the beneficial effects of aquatic PE on cognitive function, body composition and functional fitness in elderly patients. The combined PE water-based exercise group showed more beneficial effects in terms of improving cognitive function variables than the aerobic interval PE group and continuous aerobic PE group [192].

Pesce et al., demonstrated the role of myokine irisin in cognitive function during aging. Peripheral or direct irisin is generated during aerobic PE. The authors emphasized that this may be useful in the treatment of neurodegenerative diseases, as it increases cognitive performance [193].

Muller et al., concluded that PE slows down the process of neurodegeneration in the elderly, proposing it as a clinical tool for the control and treatment of Alzheimer’s disease, Parkinson’s disease and dementia. Physical and mental health is closely linked to the practice of PE and an active lifestyle. PE is a nonpharmacological therapy for the treatment of neurodegenerative diseases [194]. PE improves the condition of patients who suffer from neurodegenerative diseases by increasing the production of hormones, neurotrophic factors and neurotransmitters. PE is also responsible for optimizing neuroendocrine and physiological responses to physical and psychosocial stresses. PE promotes neuroplasticity and neuronal survival by sensitizing the nervous system through promoting processes such as neurogenesis, angiogenesis, synaptic plasticity and autophagy [195].

The ANS is a vast network of nerves affecting every organ in the body and is responsible for maintaining the balance between body and mind. Health is a result of the harmonic interchange between the SNS and PNS branches of the ANS.

**Figure 2:**
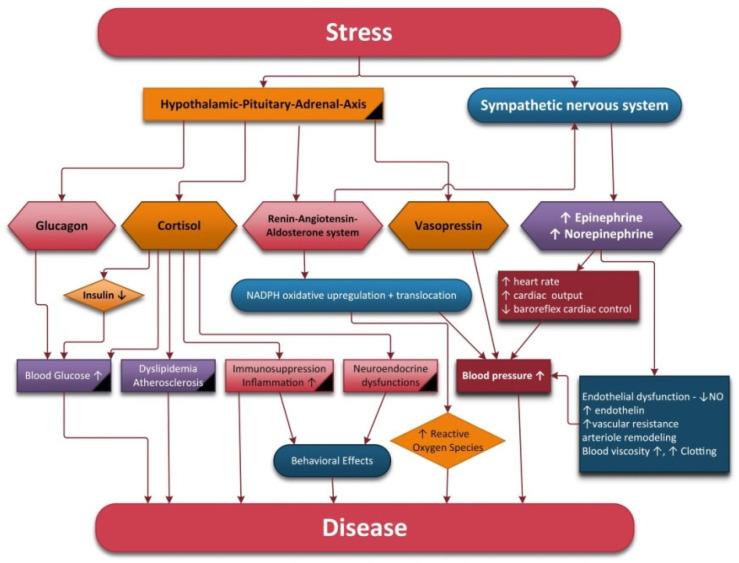
Physiological modifications under stimulation of the hypothalamic–pituitary–adrenal axis and sympathetic nervous system.

Acute stress response with predominant SNS activity is important for survival, performance and achieving various goals. However, when this activation becomes chronic it can be detrimental to our health and wellbeing. Chronic stress leads to the dysregulation of ANS, causing SNS predominance and the non-involvement of the PN. This disorder is associated with neuroendocrine, cardiovascular, respiratory, digestive and psychiatric diseases (Figure 2).

Physical exercise training is protective against cardiovascular diseases, obesity, metabolic syndrome and type 2 diabetes mellitus and is also effective to improving the performance of the autonomic nervous system.

Exercise is associated with reduced resting heart and respiratory rate and blood pressure; improved baroreflex, cardiac and endothelial function; increased skeletal muscle blood flow; and more effective redistribution of blood flow during exercise.

SNS is activated during PE but repeated physical training can reduce SNS activity and improve autonomic balance. It is generally believed that reductions in sympathetic outflow represent a major adaptation of exercise training. After exercise, slow breathing shifts the autonomic balance to parasympathetic dominance. The salutary effects of slow and deep breathing are mediated by an increase in tidal volume and the activation of the Hering–Breuer reflex, an inhibitory reflex triggered by lung stretch receptors and mediated by vagal afferents, which may increase baroreflex sensitivity [196]. In addition to stimulating the PNS, slow breathing also improves pulmonary ventilation, gas exchange and arterial oxygenation. Additionally, reduced SNS activity may be the result of a decrease in chemoreflex activity due to the reciprocal influences of the baroreflex and chemoreflex [197]. The NTS has been proposed as an integrating center for the baroreflex, chemoreflex and Hering-Breuer reflex and plays an important role in the effect of breathing on cardiovascular modulation.

The literature demonstrates a close link between oxidative stress and inflammation. Both ANS branches are able to regulate both oxidative stress and inflammation simultaneously.

During exercise, an increase in respiration and oxygen uptake take place with the purpose of directing a high quantity of O2 to the body’s vital organs. After the oxygen is used, a lot of ROS/NRS are produced. High levels of ROS induce the activation of antioxidant defenses and this will cause a positive adaptation of the nervous system. Training can decrease systemic oxidative stress and it also has a positive impact on antioxidant defenses. A single session of overload training (in large volume or over a long time period) can cause oxidative distress, leading to the loss of beneficial health outcomes related to physical activity. However, if training continues the body can adapt to the exhaustive physical activity by increasing the expression of antioxidant enzymes.

If oxidative stress is reduced or antioxidant capacity is increased with training then less inflammatory process will occur during training. Physical exercise is an efficient clinical tool that limits chronic inflammation using complex mechanisms to activate the immune system, which increases the level of anti-inflammatory cytokines and limits pro-inflammatory cytokine levels in the blood plasma and serum.

Physical activity, by enhancing the parasympathetic tone and activating the cholinergic anti-inflammatory pathway, may be a therapeutic strategy for reducing chronic inflammation and preventing many chronic diseases. If PE can produce inflammation during and after its execution, regular physical exercise training may be considered an anti-inflammatory therapy. Moreover, pro-inflammatory processes that occur after exercise may be vital for long-term adaptive responses to exercise training.

The SNS’s effects on glucose and lipid metabolism are mediated by circulating glucagon, epinephrine, direct sympathetic liver innervation, adipose tissue and skeletal muscle. The effects of the PNS on glucose and lipid metabolism are mediated by insulin and direct parasympathetic innervation of the liver. Generally, sympathetic stimulation produces catabolic effects while parasympathetic stimulation produces anabolic effects.

Sympathetic stimulation leads to hepatic glucose production by activating glycogenolysis in fed states and gluconeogenesis in fasted states. In addition, hormones, such as insulin or leptin, may indicate the peripheral metabolic state, the hypothalamus being the principal site for the integration of autonomic function in the control of appetite regulation and glucose and lipid metabolism [198]. Insulin stimulates glucose and free fatty acid uptake, inhibits lipolysis, promotes the reesterification of fatty acids to triglycerides and stimulatese lipogenesis [198]. Additionally, insulin increases protein synthesis, cell proliferation and growth and enhances SNS activity. The solitary and ambiguous nuclei in the brainstem and the dorsomedial, ventromedial, paraventricular, supraoptic and arcuate nucleii in the hypothalamus are all sensitive to insulin levels. Intracerebroventricular injection of insulin increases MSNA, which shows that insulin may act as a direct mediator of sympathetic overdrive in metabolic syndrome [199].

Several studies have shown that SNS hyperactivity precedes and predicts the appearance of impaired glucose metabolism and insulin resistance. On the other hand, hyperinsulinemia can contribute to chronic sympathetic activation. Impaired parasympathetic regulation of glucose is a risk factor for chronic hyperglycemia and insulin resistance [200]. Insulin resistance promotes ROS accumulation, DNA damage and mitochondrial and endothelial dysfunction and can also exacerbate inflammatory responses. The secretion of leptin, a hormone produced primarily by adipose tissue that acts on specific receptors in the hypothalamus to decrease appetite, is increased by insulin, corticosteroids, TNF-alpha and estrogens and is decreased by androgens, growth hormone, catecholamines and free fatty acids [201]. Leptin promotes weight loss by increasing energy expenditure through stimulation of the SNS in thermogenic brown adipose tissue and in non-thermogenic organs (heart, kidney, adrenal medulla). A deficit of leptin can lead to obesity, insulin resistance and glucose tolerance impairment. Increased plasma leptin levels found in obesity may reflect a high fat mass and partial resistance to leptin. Weight-loss via PE reduces leptin levels and raises insulin sensitivity.

**Figure 3:**
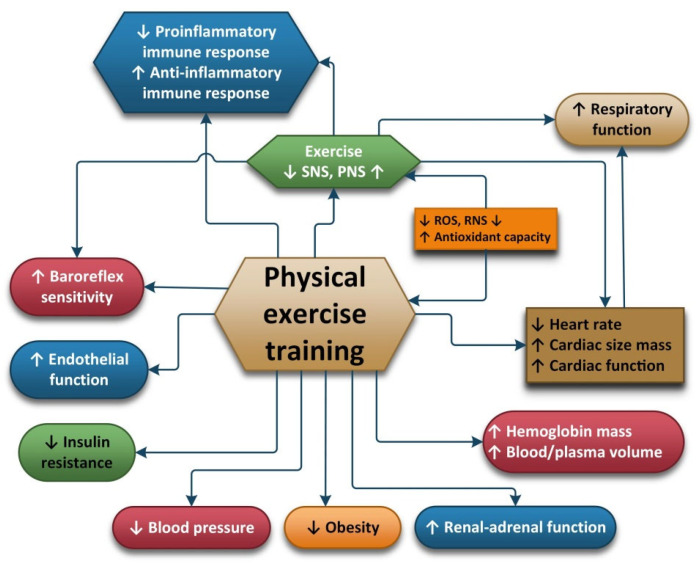
Pathophysiological changes under regular physical training.

In summary, PE causes lower HPA axis, SNS, oxidative stress and inflammatory activity and increased PNS activity. In addition, PE also contributes to greater cardiovascular and respiratory function, insulin sensitivity and neuroplasticity and higher levels of neurotrophic factors, which may all contribute to the beneficial effects of regular exercise (Figure 3).

The promotion of PE in medicine and its influence on human health has led to the assimilation of the notion of “Exercise is Medicine” [202]. At the moment, a current research topic also looks at how estrogen/progestogen secretion is influenced by physical activity [203]. 

Besides the promotion of physical exercise, having an antioxidant-rich diet with healthy eating habits can prevent oxidative stress and inflammation. The beneficial effects of endogenous antioxidants are improved by PE, but exogenous antioxidants such as vitamins, omega 3 fatty acids, polyphenols, coenzyme Q10, alpha lipoic acid, etc., can also have a positive effect on athletic performance. In recent years, many studies have been focused on the search for natural compounds that can reduce oxidative stress and inflammation. It has been observed that people consuming Mediterranean or Asian diets have a reduced risk of developing neurodegenerative diseases. It has been found that these specific diets contain high amounts of different phenolic compounds that are found in green tea, extra virgin olive oil, resveratrol, curcuminoids, fruits and aromatic herbs, which may have a preventive effect against inflammation and oxidative stress [204]. It has also been demonstrated that a diet poor in antioxidants can lead to increases in oxidative stress during intensive short-term exercise. Supplementation with multivitamins before and during a marathon has been found to prevent an increase in lipid peroxidation [205].

Taherkhani and colleagues demonstrated that increased levels of ROS and cytokines in the body act as a double-edged sword. In addition to the destructive effects of oxidative stress, they can promote processes that create various adaptations in the body, such as increasing protein synthesis, activating insulin signaling and mitochondrial biogenesis and positive regulation of antioxidants [206]. Additionally, they point out that the effect of antioxidant supplements on improving oxidative stress and inflammatory cytokines is somewhat ambiguous [205]. On the other hand, it has been shown that sulforaphane can counteract oxidative stress and suppress inflammation by decreasing NF-kB leves [207]. Similarly, terpenoids can reduce oxidative stress by stimulating the nuclear factor erythroid-2/heme oxygenase-1 pathway and to decreasing NF-kB levels [208]. Choi et al. reported that the daily consumption of 1.5 L of electrolyte-reduced water was effective at reducing measures of basal oxidative stress [209]. Cannataro et al. demonstrated that consuming a ketogenic diet and microRNAs, particularly miR-30a-5p, are correlated with antioxidant homeostasis [210]. Ruhee et al. investigated the efficacy of sulforaphane on macrophages and proved that cells previously exposed to sulforaphane displayed attenuated oxidative stress and inflammation due to reductions in nitric oxide and cytokine expression [211]. Similar antioxidant effects were observed after the administration of alpha-lipoic acid by Andreeva-Gateva [212].

PE is considered a valuable non-pharmacological therapy, but it must be included in a lifestyle strategy designed to enhance overall wellbeing, in which diet also plays an important role. This combination can beneficially influence oxidative stress and inflammation levels [213]. 

## 3. Concluding Remarks

In this review, the recent scientific literature has been carefully analyzed in order to demonstrate the existence of a close relationship between physical training and anti-inflammatory and antioxidant effects on the autonomic nervous system.

We know a lot about the benefits of exercise, but lack information about the correct mode, type, length and frequency of exercise necessary to gain such benefits. The quality of low-volume exercise performed with high intensity has become more and more noteworthy with many studies showcasing the comparable efficiencies of high-intensity interval workouts and traditional training [214].

Even though there are many ways in which physical exercise can be structured, it is important that individualized training is prescribed that takes into account the characteristics of the person doing the training with the aim of achieving the optimal physiological outcomes. Stanley et al. suggested that a monitory system should be used in which the HRV and training logs are included in the daily routine of the person doing the exercise [215]. Their results show that the time required for complete cardiac autonomic recovery after a single aerobic-based training session is up to 24 h for low-intensity exercise, 24–48 h for threshold intensity exercise, and at least 48 h following high-intensity exercise [215]. 

The recommendations regarding moderate-intensity exercises for most people are 30 min/day 5 days/week. For people with diseases like autonomic disorders, the training should be conducted under expert supervision. Moderate exercise is recommended to improve physiological and functional capabilities because it increases the expression of antioxidant enzymes. Mechanistic analyses of free radicals and antioxidants may be useful for physiotherapists and health professionals, particularly when comparing different exercise regimes in order to outline appropriate recommendations for physical exercise in health guidelines.

### Summary and Future Directions

The ANS is a vast network of nerves affecting every organ in the body and is responsible for maintaining the balance between the body and the mind. Health is a result of the harmonic interchange between the SNS and PNS branches of the ANS.

## References

[1] Benarroch E.E. (2014). Central Autonomic Network.

[2] Kanji M., Shirai M., Murata J., Tsuchimochi H., Komine H., Ninomiya I., Shimizu K. (2002). Sympathetic Cholinergic Vasodilation of Skeletal Muscle Small Arteries. Jpn. J. Pharmacol..

[3] Woolf N.J., Butcher L.L. (2011). Cholinergic systems mediate action from movement to higher consciousness. Behav. Brain Res..

[4] Séguéla P., Wadiche J., Dineley-Miller K., Dani J.A., Patrick J.W. (1993). Molecular cloning, functional properties, and distribution of rat brain alpha 7: A nicotinic cation channel highly permeable to calcium. J. Neurosci..

[5] Benarroch E.E. (1993). The central autonomic network: Functional organization, dysfunction, and perspective. Mayo Clin. Proc..

[6] Craig A.D. (2003). Interoception: The sense of the physiological condition of the body. Curr. Opin. Neurobiol..

[7] Dampney R.A., Horiuchi J. (2003). Functional organisation of central cardiovascular pathways: Studies using c-fos gene expression. Prog. Neurobiol..

[8] Feldman J.L., Mitchell G.S., Nattie E.E. (2003). Breathing: Rhythmicity, plasticity, chemosensitivity. Annu. Rev. Neurosci..

[9] Travagli R.A., Hermann G.E., Browning K.N., Rogers R.C. (2006). Brainstem circuits regulating gastric function. Annu. Rev. Physiol..

[10] Price J.L. (2005). Free will versus survival: Brain systems that underlie intrinsic constraints on behavior. J. Comp. Neurol..

[11] Morrison S.F. (1999). RVLM and raphe differentially regulate sympathetic outflows to splanchnic and brown adipose tissue. Am. J. Physiol..

[12] Dampney R.A. (1994). Functional organization of central pathways regulating the cardiovascular system. Physiol. Rev..

[13] Morrison S.F., Nakamura K., Madden C.J. (2008). Central control of thermogenesis in mammals. Exp. Physiol..

[14] Bandler R., Keay K.A., Floyd N., Price J. (2000). Central circuits mediating patterned autonomic activity during active vs. passive emotional coping. Brain Res. Bull..

[15] Corcoran A.E., Hodges M.R., Wu Y., Wang W., Wylie C.J., Deneris E.S., Richerson G.B. (2009). Medullary serotonin neurons and central CO2 chemoreception. Respir. Physiol. Neurobiol..

[16] Price C.J., Hoyda T.D., Ferguson A.V. (2008). The area postrema: A brain monitor and integrator of systemic autonomic state. Neuroscientist.

[17] Benarroch E.E. (2012). Periaqueductal gray: An interface for behavioral control. Neurology.

[18] Saper C.B. (2002). The central autonomic nervous system: Conscious visceral perception and autonomic pattern generation. Annu. Rev. Neurosci..

[19] Holstege G. (2005). Micturition and the soul. J. Comp. Neurol..

[20] Curtis A.L., Valentino R.J. (1994). Corticotropin-releasing factor neurotransmission in locus coeruleus: A possible site of antidepressant action. Brain Res. Bull..

[21] Young E.A., Abelson J.L., Cameron O.G. (2005). Interaction of brain noradrenergic system and the hypothalamic-pituitary-adrenal (HPA) axis in man. Psychoneuroendocrinology.

[22] Ennis M., Aston-Jones G. (1988). Activation of locus coeruleus from nucleus paragigantocellularis: A new excitatory amino acid pathway in brain. J. Neurosci..

[23] Siever L.J., Uhde T.W., Insel T.R., Kaye W.H., Jimerson D.C., Lake C.R., Kafka M., Targum S., Murphy D.L. (1985). Biological alterations in the primary affective disorders and other tricyclic-responsive disorders. Prog. Neuro Psychopharmacol. Biol. Psychiatry.

[24] Chan-Palay V. (1991). Alterations in the locus coeruleus in dementias of Alzheimer’s and Parkinson’s disease. Prog. Brain Res..

[25] Phelix C.F., Liposits Z., Paull W.K. (1994). Catecholamine-CRF synaptic interaction in a septal bed nucleus: Afferents of neurons in the bed nucleus of the stria terminalis. Brain Res. Bull..

[26] Cecchi M., Khoshbouei H., Javors M., Morilak D.A. (2002). Modulatory effects of norepinephrine in the lateral bed nucleus of the stria terminalis on behavioral and neuroendocrine responses to acute stress. Neuroscience.

[27] Le Doux J. (2007). The amygdala. Curr. Biol..

[28] Ulrich-Lai Y.M., Herman J.P. (2009). Neural regulation of endocrine and autonomic stress responses. Nat. Rev. Neurosci..

[29] Shin L.M., Liberzon I. (2010). The neurocircuitry of fear, stress, and anxiety disorders. Neuropsychopharmacology.

[30] Critchley H.D., Harrison N.A. (2013). Visceral influences on brain and behavior. Neuron.

[31] Cechetto D.F., Saper C.B. (1987). Evidence for a viscerotopic sensory representation in the cortex and thalamus in the rat. J. Comp. Neurol..

[32] Critchley H.D. (2005). Neural mechanisms of autonomic, affective, and cognitive integration. J. Comp. Neurol..

[33] De Vignemont F., Singer T. (2006). The empathic brain: How, when and why?. Trends Cogn. Sci..

[34] Jabbi M., Swart M., Keysers C. (2007). Empathy for positive and negative emotions in the gustatory cortex. Neuroimage.

[35] Craig A.D. (2005). Forebrain emotional asymmetry: A neuroanatomical basis?. Trends Cogn. Sci..

[36] Beissner F., Meissner K., Bär K.J., Napadow V. (2013). The autonomic brain: An activation likelihood estimation meta-analysis for central processing of autonomic function. J. Neurosci..

[37] Vogt B.A., Vogt L., Farber N.B., Bush G. (2005). Architecture and neurocytology of monkey cingulate gyrus. J. Comp. Neurol..

[38] Harrison N.A., Brydon L., Walker C., Gray M.A., Steptoe A., Critchley H.D. (2009). Inflammation causes mood changes through alterations in subgenual cingulate activity and mesolimbic connectivity. Biol. Psychiatry.

[39] Radley J.J., Arias C.M., Sawchenko P.E. (2006). Regional differentiation of the medial prefrontal cortex in regulating adaptive responses to acute emotional stress. J. Neurosci..

[40] Hoover W.B., Vertes R.P. (2007). Anatomical analysis of afferent projections to the medial prefrontal cortex in the rat. Brain Struct. Funct..

[41] Drevets W.C., Savitz J., Trimble M. (2008). The subgenual anterior cingulate cortex in mood disorders. CNS Spectr..

[42] De Pascalis V., Ray W.J., Tranquillo I., D’Amico D. (1998). EEG activity and heart rate during recall of emotional events in hypnosis: Relationships with hypnotizability and suggestibility. Int. J. Psychophysiol..

[43] Sapolsky R.M. (2000). Stress hormones: Good and bad. Neurobiol. Dis..

[44] Tasker J.G., Herman J.P. (2011). Mechanisms of rapid glucocorticoid feedback inhibition of the hypothalamic-pituitary-adrenal axis. Stress.

[45] Riebe C.J., Wotjak C.T. (2011). Endocannabinoids and stress. Stress.

[46] Culić V. (2007). Acute risk factors for myocardial infarction. Int. J. Cardiol..

[47] Dimsdale J.E. (1991). A new mechanism linking stress to coronary pathophysiology?. Circulation.

[48] Vogt C.J., Schmid-Schönbein G.W. (2001). Microvascular endothelial cell death and rarefaction in the glucocorticoid-induced hypertensive rat. Microcirculation.

[49] Reynolds R.M., Walker B.R. (2003). Human insulin resistance: The role of glucocorticoids. Diabetes Obes. Metab..

[50] Dias-Ferreira E., Sousa J.C., Melo I., Morgado P., Mesquita A.R., Cerqueira J.J., Costa R.M., Sousa N. (2009). Chronic stress causes frontostriatal reorganization and affects decision-making. Science.

[51] Heponiemi T., Keltikangas-Järvinen L., Kettunen J., Puttonen S., Ravaja N. (2004). BIS-BAS sensitivity and cardiac autonomic stress profiles. Psychophysiology.

[52] Hayashi T. (2014). Conversion of psychological stress into cellular stress response: Roles of the sigma-1 receptor in the process. Psychiatry Clin. Neurosci..

[53] Rosas-Ballina M., Ochani M., Parrish W.R., Ochani K., Harris Y.T., Huston J.M., Chavan S., Tracey K.J. (2008). Splenic nerve is required for cholinergic anti-inflammatory pathway control of TNF in endotoxemia. Proc. Natl. Acad. Sci. USA.

[54] Woody A., Figueroa W.S., Benencia F., Zoccola P.M. (2017). Stress-induced parasympathetic control and its association with inflammatory reactivity. Psychosomat. Med..

[55] Feng W., Liu H., Luo T., Liu D., Du J., Sun J., Wang W., Han X., Yang K., Guo J. (2017). Combination of IL-6 and sIL-6R differentially regulate varying levels of RANKL-induced osteoclastogenesis through NF-kB, ERK and JNK signaling pathways. Sci. Rep..

[56] Behrens M.M., Ali S.S., Dugan L.L. (2008). Interleukin-6 mediates the increase in NADPH-Oxidase in the ketamine model of schizophrenia. J. Neurosci..

[57] Streltsova L.I., Tkacheva Î.V., Plokhova E.V., Akasheva D.U., Strazhesko I.D., Dudinskaya E. (2017). Age-related changes in heart rate variability and their relation with leucocyte telomere length. Cardiovasc. Ther. Prevent..

[58] Weber C.S., Thayer J.F., Rudat M., Wirtz P.H., Zimmermann-Viehoff F., Thomas A., Perschel F.H., Arck P.C., Deter H.C. (2010). Low vagal tone is associated with impaired post stress recovery of cardiovascular, endocrine and immune markers. Eur. J. Appl. Physiol..

[59] Straub R.H., Herfarth H., Falk W., Andus T., Scholmerich J. (2002). Uncoupling of the sympathetic nervous system and the hypothalamic-pituitary-adrenal axis in inflammatory bowel disease?. J. Neuroimmunol..

[60] Maldonado-Ruiz R., Fuentes-Mera L., Camacho A. (2017). Central modulation of neuroinflammation by neuropeptides and energy-sensing hormones during obesity. BioMed Res. Int..

[61] Newton K., Dixit V.M. (2012). Signaling in innate immunity and inflammation. Cold Spring Harb. Perspect. Biol..

[62] Chobanyan-Jürgens K., Jordan J. (2015). Autonomic nervous system activity and inflammation: Good ideas, good treatments, or both?. Am. J. Physiol. Heart Circ. Physiol..

[63] Quan N., Banks W.A. (2007). Brain-immune communication pathways. Brain Behav. Immun..

[64] Tracey K.J. (2010). Understanding immunity requires more than immunology. Nat. Immunol..

[65] D’Mello C., Le T., Swain M.G. (2009). Cerebral microglia recruit monocytes into the brain in response to tumor necrosis factoralpha signaling during peripheral organ inflammation. J. Neurosci..

[66] Dinarello C.A. (1994). The biological properties of interleukin-1. Eur. Cytokine Netw..

[67] Galic M.A., Riazi K., Pittman Q.J. (2012). Cytokines and brain excitability. Front. Neuroendocrinol..

[68] Pennica D., Nedwin G.E., Hayflick J.S. (1984). Human tumor necrosis factor: Precursor structure, expression, and homology to lymphotoxin. Nature.

[69] Billiau A. (1996). Interferon gamma: Biology and role in pathogenesis. Adv. Immunol..

[70] Mantovani A., Dejana E. (1989). Cytokines as communication signals between leukocytes and endothelial cells. Immunol. Today.

[71] Baggiolini M., Dewald B., Moser B. (1997). Human chemokines: An update. Annu. Rev. Immunol..

[72] Greenberg M.J., Streiter R.M., Kunkel S.L., Danforth J.M., Laichalk L.L., McGillicuddy D.C., Standiford T.J. (1996). Neutralization of macrophage inflammatory protein-2 attenuates neutrophil recruitment and bacterial clearance in murine Klebsiella pneumonia. J. Infect. Dis..

[73] Kluth D.C., Rees A.J. (1996). Inhibiting inflammatory cytokines. Semin. Nephrol..

[74] Te Velde A., Huijbens R.J.F., de Vries J.E. (1990). IL-4 increases FcRc membrane expression and FcRc-mediated cytotoxic activity of human monocytes. J. Immunol..

[75] Joyce D.A., Gibbons D.P., Green P. (1994). Two inhibitors of inflammatory cytokine release, interleukin 10 and interleukin 4, have contrasting effects on the release of soluble p75 tumor necrosis factor receptor by cultured monocytes. Eur. J. Immunol..

[76] Bellinger D.L., Millar B.A., Perez S., Carter J., Wood C., ThyagaRajan S., Molinaro C., Lubahn C., Lorton D. (2008). Sympathetic modulation of immunity: Relevance to disease. Cell. Immunol..

[77] Sanders V.M., Munson A.E. (1985). Norepinephrine and the antibody response. Pharmacol. Rev..

[78] Calcagni E., Elenkov I. (2006). Stress system activity, innate and T helper cytokines, and susceptibility to immune-related diseases. Ann. N. Y. Acad. Sci..

[79] Greenfeld K., Avraham R., Benish M., Goldfarb Y., Rosenne E., Shapira Y., Rudich T., Ben-Eliyahu S. (2007). Immune suppression while awaiting surgery and following it: Dissociations between plasma cytokine levels, their induced production, and NK cell cytotoxicity. Brain Behav. Immun..

[80] Thaker P.H., Han L.Y., Kamat A.A., Arevalo J.M., Takahashi R., Lu C., Jennings N.B., Armaiz-Pena G., Bankson J.A., Ravoori M. (2006). Chronic stress promotes tumor growth and angiogenesis in a mouse model of ovarian carcinoma. Nat. Med..

[81] Kin N.W., Sanders V.M. (2006). It takes nerve to tell T and B cells what to do. J. Leukoc. Biol..

[82] Pongratz G., Straub R.H. (2014). The sympathetic nervous response in inflammation. Arthritis Res. Ther..

[83] Bellinger D.L., Lorton D. (2014). Autonomic regulation of cellular immune function. Auton. Neurosci. Basic Clin..

[84] Martelli D., Yao S.T., McKinley M.J., McAllen R.M. (2014). Reflex control of inflammation by sympathetic nerves, not the vagus. J. Physiol..

[85] Straub R.H. (2004). Complexity of the bi-directional neuroimmune junction in the spleen. Trends Pharmacol. Sci..

[86] Yang X., Rothman V.L., L’Heureux D.Z., Tuszynski G. (2006). Reduction of angiocidin expression in human umbilical vein endothelial cells via siRNA silencing inhibits angiogenesis. Exp. Mol. Pathol..

[87] Nilsson M.B., Langley R.R., Fidler I.J. (2005). Interleukin-6, secreted by human ovarian carcinoma cells, is a potent proangiogenic cytokine. Cancer Res..

[88] Sica A., Allavena P., Mantovani A. (2008). Cancer related inflammation: The macrophage connection. Cancer Lett..

[89] Buijs R.M., van der Vliet J., Garidou M.L., Huitinga I., Escobar C. (2008). Spleen vagal denervation inhibits the production of antibodies to circulating antigens. PLoS ONE.

[90] Gaykema R.P., Chen C.C., Goehler L.E. (2007). Organization of immune-responsive medullary projections to the bed nucleus of the stria terminalis, central amygdala, and paraventricular nucleus of the hypothalamus: Evidence for parallel viscerosensory pathways in the rat brain. Brain Res..

[91] Sternberg E.M. (2006). Neural regulation of innate immunity: A coordinated nonspecific host response to pathogens. Nat. Rev. Immunol..

[92] Berg D.K., Conroy W.G. (2002). Nicotinic alpha 7 receptors: Synaptic options and downstream signaling in neurons. J. Neurobiol..

[93] Zoli M., Pucci S., Vilella A., Gotti C. (2018). Neuronal and extraneuronal nicotinic acetylcholine receptors. Curr. Neuropharmacol..

[94] King J.R., Gillevet T.C., Kabbani N. (2017). A G protein-coupled alpha7 nicotinic receptor regulates signaling and TNF-alpha release in microglia. FEBS Open Bio..

[95] Rosas-Ballina M., Olofsson P.S., Ochani M., Valdés-Ferrer S.I., Levine Y.A., Reardon C., Tusche M.W., Pavlov V.A., Andersson U., Chavan S. (2011). Acetylcholine-synthesizing T cells relay neural signals in a vagus nerve circuit. Science.

[96] Su X., Matthay M.A., Malik A. (2009). Requisite role of the cholinergic 7 nicotinic acetylcholine receptor pathway in suppressing gram-negative sepsisinduced acute lung inflammatory injury. J. Immunol..

[97] Vida G., Peña G., Deitch E.A., Ulloa L. (2011). α7-cholinergic receptor mediates vagal induction of splenic norepinephrine. J. Immunol..

[98] Marrero M.B., Bencherif M. (2009). Convergence of alpha 7 nicotinic acetylcholine receptor-activated pathways for antiapoptosis and anti-inflammation: Central role for JAK2 activation of STAT3 and NF-kappaB. Brain Res..

[99] Park H.J., Lee P.H., Ahn Y.W., Choi Y.J., Lee G., Lee Da Chung E.S., Jin B.K. (2007). Neuroprotective effect of nicotine on dopaminergic neurons by anti-inflammatory action. Eur. J. Neurosci..

[100] Egea J., Buendia I., Parada E., Navarro E., León R., Lopez M.G. (2015). Anti-inflammatory role of microglial alpha7 nAChRs and its role in neuroprotection. Biochem. Pharmacol..

[101] Cedillo J.L., Arnalich F., Martín-Sánchez C., Quesada A., Rios J.J., Maldifassi M.C., Atienza G., Renart J., Fernández-Capitán C., García-Rio F. (2015). Usefulness of α7 nicotinic receptor messenger RNA levels in peripheral blood mono-nuclear cells as a marker for cholinergic anti inflammatory pathway activity in septic patients: Results of a pilot study. J. Infect. Dis..

[102] Borovikova L.V., Ivanova S., Zhang M., Yang H., Botchkina G.I., Watkins L.R., Abumrad N., Eaton JWTracey K.J. (2000). Vagus nerve stimulation attenuates the systemic inflammatory response to endotoxin. Nature.

[103] Bernik T.R., Friedman S.G., Ochani M., di Raimo R., Susarla S., Czura C.J., Tracey K.J. (2002). Cholinergic anti-inflammatory pathway inhibition of tumor necrosis factor during ischemia reperfusion. J. Vasc. Surg..

[104] Powers S.K., Jackson M.J. (2008). Exercise-induced oxidative stress: Cellular mechanisms and impact on muscle force production. Physiol. Rev..

[105] Montezano A.C., Touyz R.M. (2012). Reactive oxygen species and endothelial function--role of nitric oxide synthase uncoupling and Nox family nicotinamide adenine dinucleotide phosphate oxidases. Basic Clin. Pharmacol. Toxicol..

[106] Limón-Pacheco J.H., Gonsebatt M.E. (2010). The glutathione system and its regulation by neurohormone melatonin in the central nervous system. Cent. Nerv. Syst. Agents Med. Chem..

[107] Patki G., Solanki N., Atrooz F., Allam F., Salim S. (2013). Depression, anxiety-like behavior and memory impairment are associated with increased oxidative stress and inflammation in a rat model of social stress. Brain Res..

[108] Huang M.L., Chiang S., Kalinowski D.S., Bae D.H., Sahni S., Richardson D.R. (2019). The Role of the Antioxidant Response in Mitochondrial Dysfunction in Degenerative Diseases: Cross-Talk between Antioxidant Defense, Autophagy, and Apoptosis. Oxid. Med. Cell. Longev..

[109] Salim S. (2017). Oxidative Stress and the Central Nervous System. J. Pharmacol. Exp. Ther..

[110] Wang X., Michaelis E.K. (2010). Selective neuronal vulnerability to oxidative stress in the brain. Front. Aging Neurosci..

[111] Yan G., You B., Chen S., Liao J.K., Sun J. (2008). Tumor necrosis factor-α downregulates endothelial nitric oxide synthase mRNA stability via translation elongation factor 1-α 1. Circ. Res..

[112] Mäki-Petäjä K.M., Hall F.C., Booth A.D., Wallace S.M., Yasmin, Bearcroft P.W., Harish S., Furlong A., McEniery C.M., Brown J. (2006). Rheumatoid arthritis is associated with increased aortic pulse-wave velocity, which is reduced by anti-tumor necrosis factor-α therapy. Circulation.

[113] Hirooka Y., Kishi T., Sakai K., Takeshita A., Sunagawa K. (2011). Imbalance of central nitric oxide and reactive oxygen species in the regulation of sympathetic activity and neural mechanisms of hypertension. Am. J. Physiol. Regul. Integr. Comp. Physiol..

[114] Golbidi S., Ebadi S.A., Laher I. (2011). Antioxidants in the treatment of diabetes. Curr. Diabetes Rev..

[115] Harris K.F., Matthews K.A. (2004). Interactions between autonomic nervous system activity and endothelial function: A model for the development of cardiovascular disease. Psychosom. Med..

[116] Isogawa A., Yamakado M., Yano M., Shiba T. (2009). Serum superoxide dismutase activity correlates with the components of metabolic syndrome or carotid artery intima-media thickness. Diabetes Res. Clin. Pract..

[117] Lu A.L., Li X., Gu Y., Wright P.M., Chandy D.Y. (2001). Repair of oxidative DNA damage: Mechanisms and functions. Cell. Biochem. Biophys..

[118] Coluzzi E., Colamartino M., Cozzi R., Leone S., Meneghini C., O’Callaghan N., Sgura A. (2014). Oxidative stress induces persistent telomeric DNA damage responsible for nuclear morphology change in mammalian cells. PLoS ONE.

[119] Ramlee M.K., Wang J., Toh W.X., Li S. (2016). Transcription regulation of the human telomerase reverse transcriptase (hTERT) gene. Genes.

[120] Deeb D., Gao X., Liu Y., Varma N.R.S., Arbab A.S., Gautam S.C. (2013). Inhibition of telomerase activity by oleanane triterpenoid CDDO-Me in pancreatic cancer cells is ROS-dependent. Molecules.

[121] Epel E.S., Daubenmier J., Moskowitz J.T., Folkman S., Blackburn E. (2009). Can meditation slow rate of cellular aging? Cognitive stress, mindfulness, and telomeres. Ann. N. Y. Acad. Sci..

[122] Kroenke C.H., Epel E., Adler N., Bush N.R., Obradovic J., Lin J., Blackburn E., Stamperdahl J.L., Boyce W.T. (2011). Autonomic and adrenocortical reactivity and buccal cell telomere length in kindergarten children. Psychosom. Med..

[123] Banks W.A., Erickson M.A. (2010). The blood-brain barrier and immune function and dysfunction. Neurobiol. Dis..

[124] Saso L., Firuzi O. (2014). Pharmacological applications of antioxidants: Lights and shadows. Curr. Drug Targets.

[125] U.S. Department of Health and Human Services (2018). Physical Activity Guidelines for Americans.

[126] Budde H., Schwarz R., Velasques B., Ribeiro P., Holzweg M., Machado S., Brazaitis M., Staack F., Wegner M. (2016). The need for differentiating between exercise, physical activity, and training. Autoimmun. Rev..

[127] Jetté M., Sidney K., Blümchen G. (1990). Metabolic equivalents (METS) in exercise testing, exercise prescription, and evaluation of functional capacity. Clin. Cardiol..

[128] Djordjevic D., Cubrilo D., Macura M., Barudzic N., Djuric D., Jakovljevic V. (2011). The influence of training status on oxidative stress in young male handball players. Mol. Cell. Biochem..

[129] Somani S.M., Husain K., Sen C.K., Packer L., Hanninen O. (2000). Influence of exercise-induced oxidative stress on the central nervous system. Handbook of Oxidants and Antioxidants in Exercise.

[130] Vezzoli A., Pugliese L., Marzorati M., Serpiello F.R., la Torre A., Porcelli S. (2014). Time-Course Changes of Oxidative Stress Response to High-Intensity Discontinuous Training versus Moderate-Intensity Continuous Training in Masters Runners. PLoS ONE.

[131] Yen C.-J., Hung C.-H., Tsai W.-M., Cheng H.-C., Yang H.-L., Lu Y.-J., Tsai K.-L. (2020). Effect of Exercise Training on Exercise Tolerance and Level of Oxidative Stress for Head and Neck Cancer Patients Following Chemotherapy. Front. Oncol..

[132] Finkler M., Lichtenberg D., Pinchuk I. (2014). The relationship between oxidative stress and exercise. J. Basic Clin. Physiol. Pharmacol..

[133] González-Bartholin R., Mackay K., Valladares D., Zbinden-Foncea H., Nosaka K., Peñailillo L. (2019). Changes in oxidative stress, inflammation and muscle damage markers following eccentric versus concentric cycling in older adults. Eur. J. Appl. Physiol..

[134] Dantas de Lucas R., Caputob F., Mendes de Souza K., Sigwalt A.R., Ghisoni K., Silveira P.C.L., Remor A.P., Scheffer D.d.L., Guglielmo L.G.A., Latini A. (2014). Increased platelet oxidative metabolism, blood oxidative stress and neopterin levels after ultra-endurance exercise. J. Sports Sci..

[135] Tong K.T., Kong Z., Lin H., Lippi G., Zhang H., Nie J. (2013). Serum Oxidant and Antioxidant Status Following an All-Out 21-km Run in Adolescent Runners Undergoing Professional Training—A One-Year Prospective Trial. Int. J. Mol. Sci..

[136] Hendrix J., Nijs J., Ickmans K., Godderis L., Ghosh M., Polli A. (2020). The Interplay between Oxidative Stress, Exercise, and Pain in Health and Disease: Potential Role of Autonomic Regulation and Epigenetic Mechanisms. Antioxidants.

[137] Fu Q., Levine B.D. (2013). Exercise and the autonomic nervous system. Handb. Clin. Neurol..

[138] Hautala AJKiviniemi A.M., Tulppo M.P. (2009). Individual responses to aerobic exercise: The role of the autonomic nervous system. Neurosci. Biobehav. Rev..

[139] Li G., Liu J.-Y., Zhang H.-X., Li Q., Zhang S.W. (2015). Exercise Training Attenuates Sympathetic Activation and Oxidative Stress in Diet-Induced Obesity. Physiol. Res..

[140] Conti F.F., de Oliveira Brito J., Bernardes N., Dias D.d.S., Malfitano C., Morris M., Llesuy S.F., Irigoyen M.-C., de Angelis K. (2015). Positive effect of combined exercise training in a model of metabolic syndrome and menopause: Autonomic, inflammatory, and oxidative stress evaluations. Am. J. Physiol. Regul. Integr. Comp. Physiol..

[141] Cikrikcioglu M.A., Hursitoglu M., Erkal H., Kınas B.E., Sztajzel J., Cakirca M., Arslan A.G., Erek A., Halac G., Tukek T. (2011). Oxidative stress and autonomic nervous system functions in restless legs syndrome. Eur. J. Clin. Investig..

[142] Polli A., Van Oosterwijck J., Nijs J., Marusic U., De Wandele I., Paul L., Meeus M., Moorkens G., Lambrecht L., Ickmans K. (2019). Relationship Between Exercise-induced Oxidative Stress Changes and Parasympathetic Activity in Chronic Fatigue Syndrome: An Observational Study in Patients and Healthy Subjects. Clin. Ther..

[143] Cobley J.N. (2020). How exercise induces oxidative eustress. Oxidative Stress.

[144] Pedersen B.K., Ostrowski K., Rohde T., Bruunsgaard H. (1998). The cytokine response to strenuous exercise. Can. J. Physiol. Pharmacol..

[145] Pedersen M., Lexell J., Deierborg T. (2015). Effects of physical exercise on neuroinflammation, neuroplasticity, neurodegeneration, and behavior. Neurorehabil. Neural Repair.

[146] Fischer C.P., Hiscock N., Basu S., Vessby B., Kallner A., Sjöberg L.B., Febbraio M.A., Pedersen B.K. (2004). Supplementation with vitamins C and E inhibits the release of interleukin-6 from contracting human skeletal muscle. J. Physiol..

[147] Pedersen B.K., Steensberg A., Schjerling P. (2001). Muscle-derived interleukin-6: Possible biological effects. J. Physiol..

[148] Mathur N., Pedersen B.K. (2009). Exercise as a mean to control low-grade systemic inflammation. Mediat. Inflamm..

[149] LuzScheffer D., Latini A. (2020). Exercise-induced immune system response: Anti-inflammatory status on peripheral and central organs. Biochim. Biophys. Acta BBA—Mol. Basis Dis..

[150] Ostrowski K., Rohde T., Zacho M., Asp S., Pedersen B.K. (1998). Evidence that interleukin-6 is produced in human skeletal muscle during prolonged running. J. Physiol..

[151] Pedersen B.K., Febbraio M.A. (2008). Muscle as an endocrine organ: Focus on muscle-derived interleukin 6. Physiol. Rev..

[152] Alizaei Yousefabadi H., Niyazi A., Alaee S., Fathi M., Mohammad Rahimi G.R. (2020). Anti-Inflammatory Effects of Exercise on Metabolic Syndrome Patients: A Systematic Review and Meta-Analysis. Biol. Res. Nurs..

[153] Balducci S., Zanuso S., Nicolucci A., Fernando F., Cavallo S., Cardelli P., Fallucca S., Alessi E., Letizia C., Jimenez A. (2010). Anti-inflammatory effect of exercise training in subjects with type 2 diabetes and the metabolic syndrome is dependent on exercise modalities and independent of weight loss. Nutr. Metab. Cardiovasc. Dis..

[154] Dadrass A., Mohamadzadeh Salamat K., Hamidi K., Azizbeigi K. (2019). Anti-inflammatory effects of vitamin D and resistance training in men with type 2 diabetes mellitus and vitamin D deficiency: A randomized, double-blinded, placebo-controlled clinical trial. J. Diabetes Metab. Disord..

[155] Starkie R., Ostrowski S.R., Jauffred S., Febbraio M., Pedersen B.K. (2003). Exercise and IL-6 infusion inhibit endotoxin-induced TNF-alpha production in humans. FASEB J..

[156] Rosenbaum M., Nonas C., Weil R., Horlick M., Fennoy I., Vargas I., Kringas P. (2007). Camino Diabetes Prevention Group. School-based intervention acutely improves insulin sensitivity and decreases inflammatory markers and body fatness in junior high school students. J. Clin. Endocrinol. Metab..

[157] Salamat K.M., Azarbayjani M.A., Yusof A., Dehghan F. (2016). The response of pre-inflammatory cytokines factors to different exercises (endurance, resistance, concurrent) in overweight men. Alex. J. Med..

[158] Chen C.-W., Kuo Y.-C., How C.-K., Juan C.-C. (2020). Long-term aerobic exercise training-induced anti-inflammatory response and mechanisms: Focusing on the toll-like receptor 4 signaling pathway. Chin. J. Physiol..

[159] Pedersen B.K., Rohde T., Zacho M. (1996). Immunity in athletes. J. Sports Med. Phys. Fit..

[160] Goh J., Lim C.L., Suzuki K., Schumann M., Rønnestad B.R. (2019). Effects of Endurance, Strength, and Concurrent Training on Cytokines and Inflammation. Concurrent Aerobic and Strength Training.

[161] Pedersen B.K., Ullum H. (1994). NK cell response to physical activity: Possible mechanisms of action. Med. Sci. Sports Exerc..

[162] Cooper D.M., Radom-Aizik S., Schwindt C., Zaldivar F. (2007). Dangerous exercise: Lessons learned from dysregulated inflammatory responses to physical activity. J. Appl. Physiol..

[163] Tidball J.G. (2005). Inflammatory processes in muscle injury and repair. J. Physiol. Regul. Integr. Comp. Physiol..

[164] Suzuki K., Nakaji S., Yamada M., Totsuka M., Sato K., Sugawara K. (2002). Systemic inflammatory response to exhaustive exercise. Cytokine kinetics. Exerc. Immunol. Rev..

[165] Suzuki K. (2019). Chronic inflammation as an immunological abnormality and effectiveness of exercise. Biomolecules.

[166] Rokitzki L., Logemann E., Sagredos A.N., Murphy M., Wetzel-Roth W., Keul J. (1994). Lipid peroxidation and antioxidative vitamins under extreme endurance stress. Acta Physiol. Scand..

[167] Dufaux B., Order U. (1989). Plasma elastase-1-antitrypsin, neopterin, tumor necrosis factor, and soluble interleukin-2 receptor after prolonged exercise. Int. J. Sports Med..

[168] Moldoveanu A.I., Shephard R.J., Shek P.N. (2001). The cytokine response to physical activity and training. Sports Med..

[169] Fischer C.P. (2006). Interleukin-6 in acute exercise and training: What is the biological relevance?. Exerc. Immunol. Rev..

[170] Hellsten Y., Frandsen U., Orthenblad N., Sjodin N., Richter E.A. (1997). Xanthine oxidase in human skeletal muscle following eccentric exercise: A role of inflammation. J. Physiol..

[171] Nybo L., Nielsen B., Pedersen B.K., Moller K., Secher N.H. (2002). Interleukin-6 release from the human brain during prolonged exercise. J. Physiol..

[172] Van Wagoner N.J., Benveniste E.N. (1999). Interleukin-6 expression and regulation in astrocytes. J. Neuroimmunol..

[173] Sung Y.-H., Kim S.-C., Hong H.-P., Park C.-Y., Shin M.-S., Kim C.-J., Seo J.-H., Kim C.-Y., Kim D.-J., Cho H.-J. (2012). Treadmill exercise ameliorates dopaminergic neuronal loss through suppressing microglial activation in Parkinson’s disease mice. Life Sci..

[174] Duman C.H., Schlesinger L., Terwilliger R., Russell D.S., Newton S.S., Duman R.S. (2009). Peripheral insulin-like growth factor-I produces antidepressant-like behavior and contributes to the effect of exercise. Behav. Brain Res..

[175] Qian L., Wu H.M., Chen S.H., Zhang D., Ali S.F., Peterson L., Wilson B., Lu Ru Hong J.-S., Flood P.M. (2011). Beta2-adrenergic receptor activation prevents rodent dopaminergic neurotoxicity by inhibiting microglia via a novel signaling pathway. J. Immunol..

[176] Fragala M.S., Kraemer W.J., Mastro A.M., Denegar C.R., Volek J.S., Häkkinen K., Anderson J.M., Lee E.C., Maresh C.M. (2011). Leukocyte beta2-adrenergic receptor expression in response to resistance exercise. Med. Sci. Sports Exerc..

[177] Fry A.C., Schilling B.K., Weiss L.W., Chiu L.Z. (2006). Beta2-adrenergic receptor downregulation and performance decrements during high-intensity resistance exercise overtraining. J. Appl. Physiol..

[178] Radom-Aizik S., Zaldivar F.P., Haddad F., Cooper D.M. (2014). Impact of brief exercise on circulating monocyte gene and microRNA expression: Implications for atherosclerotic vascular disease. Brain Behav. Immun..

[179] Monje M.L., Toda H., Palmer T.D. (2003). Inflammatory blockade restores adult hippocampal neurogenesis. Science.

[180] Schmolesky M.T., Webb D.L., Hansen R.A. (2013). The effects of aerobic exercise intensity and duration on levels of brain-derived neurotrophic factor in healthy men. J. Sports Sci. Med..

[181] Pluchino N., Russo M., Santoro A.N., Litta P., Cela V., Genazzani A.R. (2013). Sterioid hormones and BDNF. Neuroscience.

[182] Smyth D.G. (2016). 60 years of POMC: Lipotropin and beta-endorphin: A perspective. J. Mol. Endocrinol..

[183] Hawkes C.H. (1992). Endorphins: The basis of pleasure?. J. Neurol. Neurosurg. Psychiatry.

[184] Schwarz L., Kindermann W. (1992). Changes in beta-endorphin levels in response to aerobic and anaerobic exercise. Sports Med..

[185] Siswantoyo, Aman M.S. (2014). The Effects of Breathing Exercise Toward IgG, Beta Endorphin and Blood Glucose Secretion. Asia Pac. J. Educ. Arts Sci..

[186] Dietrich A., McDaniel W.F. (2004). Endocannabinoids and exercise. Br. J. Sports Med..

[187] Maccarrone M. (2017). Metabolism of the Endocannabinoid Anandamide: Open Questions after 25 Years. Front. Mol. Neurosci..

[188] Sparling P.B., Giuffrida A., Piomelli D., Rosskopf L., Dietrich A. (2003). Exercise activates the endocannabinoid system. Neuroreport.

[189] Stensson N., Gerdle B., Ernberg M., Mannerkorpi K., Kosek E., Ghafouri B. (2020). Increased Anandamide and Decreased Pain and Depression after Exercise in Fibromyalgia. Med. Sci. Sports Exerc..

[190] Barbosa F.J., dos Lacerda J.R.M., Cristina-Souza G., Lopes Filho B.J.P., Camilo B.d.F. (2021). Effect of resistance training in women with fibromyalgia: A review study. Res. Soc. Dev..

[191] Marin Bosch B., Bringard A., Logrieco M.G., Lauer E., Imobersteg N., Thomas A., Ferretti G., Schwartz S., Igloi K. (2020). Effect of acute physical exercise on motor sequence memory. Sci. Rep..

[192] Thomas R., Johnsen L.K., Geertsen S.S., Christiansen L., Ritz C., Roig M., Lundbye-Jensen J. (2016). Acute Exercise and Motor Memory Consolidation: The Role of Exercise Intensity. PLoS ONE.

[193] Farinha C., Teixeira A.M., Serrano J., Santos H., Campos M.J., Oliveiros B., Silva F.M., Cascante-Rusenhack M., Luís P., Ferreira J.P. (2021). Impact of Different Aquatic Exercise Programs on Body Composition, Functional Fitness and Cognitive Function of Non-Institutionalized Elderly Adults: A Randomized Controlled Trial. Int. J. Environ. Res. Public Health.

[194] Pesce M., La Fratta I., Paolucci T., Grilli A., Patruno A., Agostini F., Bernetti A., Mangone M., Paoloni M., Invernizzi M. (2021). From Exercise to Cognitive Performance: Role of Irisin. Appl. Sci..

[195] Muller P., Taubert M., Muller N.G. (2019). Physical exercise as personalized medicine for dementia prevention?. Front. Physiol..

[196] Mahalakshmi B., Maurya N., Lee S.D., Bharath Kumar V. (2020). Possible Neuroprotective Mechanisms of Physical Exercise in Neurodegeneration. Int. J. Mol. Sci..

[197] Zou Y., Zhao X., Hou Y.Y., Liu T., Wu Q., Huang Y.H., Wang X.H. (2017). Meta-analysis of effects of voluntary slow breathing exercises for control of heart rate and blood pressure in patients with cardiovascular diseases. Am. J. Cardiol..

[198] Bernardi L., Porta C., Spicuzza L., Bellwon J., Spadacini G., Frey A.W., Yeung L.Y.C., Sanderson J.E., Pedretti R., Tramarin R. (2002). Slow breathing increases arterial baroreflex sensitivity in patients with chronic heart failure. Circulation.

[199] Straznicky N.E., Nestel P.J., Esler M. (2010). Autonomic Nervous System: Metabolic Function. Encycl. Neurosci..

[200] Johnson M.S., DeMarco V.G., Whaley-Connell A., Sowers J.R. (2012). Insuline resistance and the autonomic nervous system. Primer on the Autonomic Nervous System.

[201] Robertson D., Biaggioni I., Burnstock G., Low P.A., Paton J.F.R. (2012). Primer on the Autonomic Nervous System.

[202] Borer K.T. (2014). Counter regulation of insulin by leptin as key component of autonomic regulation of body weight. World J. Diabetes.

[203] Rocha-Rodrigues S., Sousa M., Lourenço Reis P., Leão C., Cardoso-Marinho B., Massada M., Afonso J. (2021). Bidirectional Interactions between the Menstrual Cycle, Exercise Training, and Macronutrient Intake in Women: A Review. Nutrients.

[204] Roberts L., Suzuki K. (2019). Exercise and Inflammation. Antioxidants.

[205] Machefer G., Groussard C., Vincent S., Zouhal H., Faure H., Cillard J., Radák Z., Gratas Delamarche A. (2007). Multivitamin-mineral supplementation prevents lipid peroxidation during “the Marathon des Sables”. J. Am. Coll. Nutr..

[206] Taherkhani S., Suzuki K., Castell L. (2020). A Short Overview of Changes in Inflammatory Cytokines and Oxidative Stress in Response to Physical Activity and Antioxidant Supplementation. Antioxidants.

[207] Ruhee R.T., Suzuki K. (2020). The Integrative Role of Sulforaphane in Preventing Inflammation, Oxidative Stress and Fatigue: A Review of a Potential Protective Phytochemical. Antioxidants.

[208] Proshkina E., Plyusnin S., Babak T., Lashmanova E., Maganova F., Koval L., Platonova E., Shaposhnikov M., Moskalev A. (2020). Terpenoids as Potential Geroprotectors. Antioxidants.

[209] Choi Y.A., Lee D.H., Cho D.-Y., Lee Y.-J. (2020). Outcomes Assessment of Sustainable and Innovatively Simple Lifestyle Modification at theWorkplace-Drinking Electrolyzed-ReducedWater (OASIS-ERW): A Randomized, Double-Blind, Placebo-Controlled Trial. Antioxidants.

[210] Cannataro R., Caroleo M.C., Fazio A., La Torre C., Plastina P., Gallelli L., Lauria G., Cione E. (2019). Ketogenic Diet and microRNAs Linked to Antioxidant Biochemical Homeostasis. Antioxidants.

[211] Ruhee R.T., Ma S., Suzuki K. (2019). Sulforaphane Protects Cells against Lipopolysaccharide-Stimulated Inflammation in Murine Macrophages. Antioxidants.

[212] Andreeva-Gateva P., Traikov L., Sabit Z., Bakalov D., Tafradjiiska-Hadjiolova R. (2020). Antioxidant Effect of Alpha-Lipoic Acid in 6-Hydroxydopamine Unilateral Intrastriatal Injected Rats. Antioxidants.

[213] Roberts L.A., Suzuki K. (2021). Anti-Inflammatory and Antioxidant Effects of Dietary Supplementation and Lifestyle Factors. Antioxidants.

[214] Steckhan N., Hohmann C.D., Kessler C., Dobos G., Michalsen A., Cramer A. (2016). Effects of different dietary approaches on inflammatory markers in patients with metabolic syndrome: A systematic review and meta-analysis. Nutrition.

[215] Stanley J., Peake J.M., Buchheit M. (2013). Cardiac parasympathetic reactivation following exercise: Implications for training prescription. Sports Med..

